# Population correlates of rapid captive‐induced maladaptation in a wild fish

**DOI:** 10.1111/eva.12649

**Published:** 2018-06-19

**Authors:** Dylan J. Fraser, Lisa Walker, Matthew C. Yates, Kia Marin, Jacquelyn L. A. Wood, Thais A. Bernos, Carol Zastavniouk

**Affiliations:** ^1^ Department of Biology Concordia University Montreal QC Canada; ^2^ Institute of Parasitology McGill University Montreal QC Canada; ^3^ Golder Associés Ltée Montréal QC Canada; ^4^ Ontario Ministry of Natural Resources and Forestry Peterborough ON Canada; ^5^ Professionals for Fair Development Protected Areas Program Paris France; ^6^ Golder Associates Limited Calgary AB Canada

**Keywords:** adaptation, adaptive potential, brook trout, captive breeding, captivity, effective population size, phenotypic plasticity, salmonid

## Abstract

Understanding the extent to which captivity generates maladaptation in wild species can inform species recovery programs and elucidate wild population responses to novel environmental change. Although rarely quantified, effective population size (*N*_*e*_) and genetic diversity should influence the magnitude of plastic and genetic changes manifested in captivity that reduce wild fitness. Sexually dimorphic traits might also mediate consequences of captivity. To evaluate these relationships, we generated >600 full‐ and half‐sibling families from nine wild brook trout populations, reared them for one generation under common, captive environmental conditions and contrasted several fitness‐related traits in wild versus captive lines. We found substantial variation in lifetime success (lifetime survival and reproductive success) and life history traits among wild populations after just one captive generation (fourteen‐ and threefold ranges across populations, respectively). Populations with lower heterozygosity showed lower captive lifetime success, suggesting that captivity generates maladaptation within one generation. Greater male‐biased mortality in captivity occurred in populations having disproportionately higher growth rates in males than females. Wild population *N*_*e*_ and allelic diversity had little or no influence on captive trait expression and lifetime success. Our results have four conservation implications: (i) Trait values and lifetime success were highly variable across populations following one generation of captivity. (ii) Maladaptation induced by captive breeding might be particularly intense for the very populations practitioners are most interested in conserving, such as those with low heterozygosity. (iii) Maladaptive sex differences in captivity might be associated with population‐dependent growth costs of reproduction. (iv) Heterozygosity can be a good indicator of short‐term, intraspecific responses to novel environmental change.

## 1 INTRODUCTION

Environmental conditions and hence, selective pressures, differ between captive and wild environments such that captivity routinely causes phenotypic changes that influence fitness in the wild (Frankham, 2008). As wild trait expression is locally adaptive in many species (Hereford, 2009), deviations from wild‐type trait values in captive‐reared individuals often result in maladaptation when they are released into nature as part of species restoration programs (Frankham, 2008). Specifically, the selective environment in captivity can favour traits that perform well in captivity, but reduce fitness in the wild relative to wild‐type traits. Such maladaptive changes can occur within one or two captive generations (Araki, Cooper & Blouin, 2007; Christie, Marine, French & Blouin, 2012); phenotypic changes within single generations can also have carry‐over effects on wild fitness (Araki, Cooper & Blouin, 2009; Evans, Wilke, O'Reilly & Fleming, 2014).

Less is known regarding how the severity and manner by which phenotypic change accrued from captive exposure differs across intraspecific populations (Fraser, 2008; Reisenbichler, 2004; Woodworth, Montgomery, Driscoe & Frankham, 2002). Such information would be useful for informing species conservation programs. As anthropogenic influences increase, many species exist as fragmented populations that differ from one another in terms of phenotypic characteristics and levels of genetic diversity. If certain genetic and phenotypic attributes influence captivity‐mediated maladaptation, conservation programs utilizing captive breeding and rearing could be improved. First, such programs could make more informed decisions regarding risks associated with captivity, for example, by revising breeding designs to minimize the risk of maladaptation, thereby increasing the likelihood of success. Second, conservation groups could better forecast which populations might demographically benefit from supplementation while minimizing genetic risks. Not only would this increase the likelihood that species recovery programs achieve their desired goals, but it would help, more generally, with setting conservation priorities within a given species.

The extent of plastic/genetic changes from captive exposure (and hence the extent of maladaptation) might be influenced by a wild population's effective population size (*N*_*e*_) and standing level of genetic diversity. Populations with low *N*_*e*_ and/or low genetic diversity, often the focus of captive breeding and supplementation, can respond to selection less effectively than large *N*_*e*_ populations (Robertson, 1960; Weber & Diggins, 1990). Theoretically, reduced selective responses in small *N*_*e*_ or low diversity populations should help to minimize genetic changes in captivity (Woodworth et al., 2002). Yet the evolution of some small *N*_*e*_ populations in nature is still heavily influenced by selection (Benazzo et al., 2017; Fraser, Debes, Bernatchez & Hutchings, 2014; Funk et al., 2016; Wood, Yates & Fraser, 2016), meaning that some adaptation to captivity is still likely. Furthermore, a captive environment will be invariably novel for any wild population initially, with two possible consequences in short‐term (within‐generation) conservation programs. First, captive mortality of wild individuals can be high (Fraser, 2016): large and small *N*_*e*_ populations might both experience viability selection, although perhaps disproportionately more will occur in populations with low *N*_*e*_ or low diversity. Second, novel environmental conditions can result in the differential phenotypic expression of neutral genetic diversity (Ghalambor, MacKay, Carroll & Reznick, 2007; Schlichting, 2008). The degree of such novel, plastic expression is expected to positively correlate with *N*_*e*_ and thus allow larger wild populations to plastically tolerate captive conditions, but it may also generate strongly maladaptive phenotypes when captive‐reared individuals are released into nature. Collectively, these points suggest that captivity may elicit a variety of plastic and genetic changes in populations of varying levels of genetic diversity and *N*_*e*_.

Sexual‐selective pressures also influence differences in phenotypic trait expression between males and females within wild populations (Langerhans & Dewitt, 2004). Sexually dimorphic traits might therefore mediate the fitness consequences of being in captivity and affect each sex in different ways. Indeed, such traits are often associated with reproductive success in the wild, reproductive investment in general and/or postreproductive survival (Ford, Murdoch & Howard, 2012; Hutchings, 2006). Given that sexual‐selective pressures vary among intraspecific populations in relation to local environmental features (Zastavniouk, Weir & Fraser, 2017), plastic and genetic changes to male and female phenotypes in the captive environment may often be population‐dependent.

Here, we aim to elucidate the potential effects of genetic and trait diversity of wild populations on captivity‐mediated fitness consequences. We are unaware of any examinations of the extent to which captivity generates plastic and genetic changes across a large number of wild populations, and specifically how these changes might relate to wild population attributes. Our examination is based on nine stream populations of brook trout, *Salvelinus fontinalis* (Mitchill 1814) from Cape Race, NL, Canada (Supporting Information Figure S1). The remarkable phenotypic diversity and local adaptation among populations within this species and related salmonid fishes are recognized for their import to species’ persistence and productivity (Fraser, Weir, Bernatchez, Hansen & Taylor, 2011; Schindler et al., 2010). Native salmonid populations, including brook trout, are experiencing unprecedented population declines in several geographic regions and are the focus of many rehabilitation and restoration efforts (COSEWIC 2010; Eastern Brook Trout Joint Venture 2005; Myers et al., 2004; Naish et al., 2008). These declines, along with these species’ great socio‐economic importance, have led to extensive captive rearing of salmonids with billions of individuals released annually (Gozlan, Britton, Cowx & Copp, 2010; Naish et al., 2008).

Although Cape Race trout are not the focus of a conservation program, they are an exemplar model for exploring the extent of maladaptation to captivity. The populations are virtually pristine and highly differentiated in morphology and life history in a manner consistent with an adaptive basis, despite occurring at a fine geographic scale (15 km × 10 km; Hutchings, 1993; Fraser et al., 2014; Wood, Tezel, Joyal & Fraser, 2015; Zastavniouk et al., 2017). Cape Race populations also vary substantially in *N*_*e*_ (Bernos & Fraser, 2016) and most have been isolated for thousands of years (Danzmann, Morgan, Jones, Bernatchez & Issen, 1998). Finally, due to their close proximity and relatively small body size, many populations can be compared under common captive conditions (Wood & Fraser, 2015), which is typically a challenge for vertebrates.

We generated a total of 13 to 131 families within each of the nine trout populations (mean = 73 families) to contrast their phenotypic changes and survival in a common hatchery environment. Across populations, we specifically compared (i) growth up to maturation in adults and female reproductive investment; (ii) deviations in growth and reproductive investment of mature captive‐born versus wild females within populations, as a metric of maladaptation from captivity; (iii) mean survival of captive‐born juveniles from egg to the first year (replicated in two different experimental years); and (iv) mean survival from egg to yolk absorption of progeny generated from captive‐born parents. We further investigated potential correlates of the severity of phenotypic/genetic changes incurred among wild populations in captivity, specifically contemporary *N*_*e*_ and standing levels of genetic diversity.

## 2 MATERIALS AND METHODS

### 2.1 Wild population sampling

Nine Cape Race trout populations were monitored for sexually mature individuals via electrofishing in October 2010 and 2011 (Supporting Information Figure S1 reports population names corresponding to acronyms used; Supporting Information Figure S2 is a schematic overview of the study design). Sexually mature individuals were determined from visual inspection of spawning “readiness”: a release of sperm for males and an elongated cloaca/soft belly for females with obvious egg bulges. Seven populations (excluding DY, BF) were monitored again in October 2014, along with one new population (UC). All study populations are genetically distinct according to F_ST_ using microsatellite loci (average *F*_ST_ = 0.25, range = 0.03–0.54; Wood, Belmar‐Lucero, Hutchings & Fraser, 2014); most are genetically distinct according to Q_ST_ for fifteen early behavioural, morphological and life history traits (average *Q*_ST_ = 0.36, range = 0.04–0.87; Wood et al., 2015). Most study populations are isolated by inhabiting streams that terminate as 30–50 m waterfalls into the sea, exceptions being the pairs BF–WN and DY–UO for which occasional gene flow occurs (Wood et al., 2014). Mature adults were gathered and placed in flow‐through cages within the stream channel until gamete collections the same evening (see Supporting Information Table S1 for total adults used). Gametes were then air‐shipped to Montreal in refrigerated coolers.

### 2.2 Wild population rearing in captivity

#### 2.2.1 Fertilized egg to postyolk absorption

Egg fertilization took place 10–14 hours after collection. Each wild female's eggs were subdivided into 2–7 lots; each lot was mixed with sperm from a different wild male of the same population. The 2010 crosses experienced 100% mortality due to an equipment malfunction during winter; however, 2010 data for wild female body size, fecundity, egg diameter and gonadosomatic index (GSI) were included in population comparisons to increase statistical power. Crossing yielded 389 and 265 families (half‐ and full‐sibs) in 2011 and 2014, respectively, or 43.1/33.1 families per population per year (Supporting Information Table S1). Mean family size was 20.0 eggs ± 8.0 *SD* as Cape Race females are small with low fecundity relative to other salmonids and brook trout populations (e.g., Belmar‐Lucero et al., 2012 vs. Serezli, Guzel & Kocabas, 2010). Families were incubated separately within 5.2‐cm‐diameter mesh‐bottom containers placed randomly within a single 1,000‐L recirculating tank and maintained at 6.9–7.0°C throughout incubation; see Wood et al. (2015) for more details.

#### 2.2.2 Postyolk absorption to age 12 months

In 2011 and 2014, after yolk absorption, we created two sets of 12 pooled families for each of WN, UO, FW, WC and STBC. The same number of families per population was used to standardize genetic variation among populations/year. Each within‐population set had equal numbers of individuals per family and the same 12 mothers but largely different fathers (Supporting Information Table S1; total *n* = 120), except for WC and STBC in 2014 where the sets had fewer mothers (10, 7). Each set was randomly assigned and reared in a separate, 130‐L tank (Supporting Information Figure S2). Sets were created in 2011 only for CC, DY and BF because either no eggs (DY, BF) or insufficient females (CC) were collected in 2014; DY sets were each limited to six families as only six wild females were sampled. For BC in 2011, there were only enough individuals for one set (one tank).

These captive‐born fish were raised for one year under common flow, temperature, pH (8.09 ± 0.030 *SD*), oxygenation (11.75 ± 0.15) and ad libitum feeding regimes. Tank temperatures fluctuated seasonally between 3 and 19°C (among tank temperatures were always within 0.1–0.2°C). Differential population mortality occurred over time, so tank densities were re‐standardized by removing fish from higher density tanks.

All captive‐born STBC fish (except eight in 2014) died within 6–8 weeks postyolk absorption, despite active feeding and early growth; some mortalities showed signs of an undetermined dorsal–caudal infection. The mortality in STBC tanks freed up tank space for other populations. To increase statistical power for population comparisons using 2011 crosses, we added one more tank replicate from each of two populations for which we had more fish (CC, WN; i.e., three tanks instead of two, mix of the same families above); these were included in analyses up to age 12 months and had been reared in a subdivided 1,000‐L rectangular tank under the same densities/conditions.

#### 2.2.3 Age 12 to 18 months (maturation), 2011 crosses

At age 12 months, captive‐born fish were transferred to six, larger cylindrical tanks (2200L, *n* = 175 per tank) to prevent overcrowding as trout grew and to maintain standardized tank densities. Populations were separated by tank except for BF, which shared tanks with DY and WC to maintain equal densities across populations, while remaining BC fish were reared with FW (Supporting Information Figure S2); adipose fin clips were used to discern between populations within tanks. To reduce possible tank effects, trout in each tank were rotated among tanks monthly; the six tanks were also maintained with the same conditions as in year 1.

#### 2.2.4 Separate family rearing: postyolk absorption to age 7 months, 2014 crosses

We also reared a subset of individual captive‐born families in 50, 12.5‐L tanks to quantify family‐level survival (one family per tank; 4–8 families/population, eight populations). Fish were raised for 7 months under common environmental/feeding conditions (same as pooled family rearing) and initial densities.

### 2.3 Wild population trait differentiation in captivity

#### 2.3.1 Body size to age 12 months, 2011 crosses

Fork length (mm) was measured at 0, 3, 7 and 12 months and mass (±0.1 g) at 3, 7 and 12 months. A random sampling of a range of averages of 48 to 93 fish per tank was measured across time periods. Length/mass data were analysed using linear mixed‐effects models with the *nlme* package (Pinheiro, Bates, DebRoy, Sarkar, & RC Team‐R software, 2017) in R (R Core Team 2016). Population and time period were included as, respectively, a categorical fixed effect and a continuous fixed covariate. Inclusion of a population × time period interaction term permitted calculation of population‐level growth rates; tank and tank × time period were also included as random effects. Mass was ln‐transformed; the slope of mass over time represented specific growth rate. Accounting for time‐associated heteroscedasticity and modelling separate residual variances for each population and time period provided significantly improved model fit (likelihood ratio tests (LRTs); χ^2 ^= 4920.7, *p *<* *0.001 and χ^2 ^= 143.8, *p *<* *0.001, respectively). All fixed effects and residual variance terms were tested under backwards stepwise model selection using LRTs with interaction terms tested first. All random effects were retained regardless of significance due to the experimental design. Mean growth rates and body sizes at each time period were calculated for each population using *lsmeans* (Lenth, 2015) and compared using *t* tests. *p*‐values for between‐population comparisons of growth rate and body size were Bonferroni adjusted (Rice, 1989) for each trait and time period (for body size) independently. Sex was not accounted for in analyses up to and including age 12 months because small fish size precluded confident sexing in many instances, no fish were observed with eggs and only 6.7% of discernible males within populations expressed sperm in 2012.

#### 2.3.2 Body size by sex at age 18 months, 2011 crosses

By October and November 2013, 99.4% and 96.8% of captive‐born males and females were sexually mature, respectively. Given unequal variance among populations, we analysed data using generalized least squares regressions in *nlme*. Population, sex and population × sex were included as categorical fixed effects; modelling separate residual variances per population also significantly improved model fit (LRT; χ^2 ^= 35.6, *p *<* *0.001). Model terms were tested using *F* tests under backward stepwise selection; *lsmeans* was used to compare population and sex level means, with *t* tests employed to compare population means. When population × sex was significant, comparisons were limited to between‐sex comparisons within each population or between‐population comparisons within each sex for calculating Bonferroni‐corrected significance levels (we were not interested in between‐population body size comparisons of opposite sexes).

#### 2.3.3 Female reproductive investment, 2011 crosses

We quantified fecundity, egg diameter and GSI for mature captive‐born females in November 2013. Females were anesthetized prior to removing eggs; digital photographs were taken of eggs in Petri dishes with known size standards. Fecundity (total number of eggs) per female was counted using ImageJ software (v.1.48v; Rasband, 2014). Mean egg diameter of 15 randomly selected eggs per female was measured as a proxy for egg size. GSI was calculated as the ratio of egg mass to body mass. The mean number of captive‐born females per population processed was 44 (total = 354 across populations, 71.4% of all females). This suite of traits associated with female reproductive investment measured on captive‐born females was also analysed on wild females; the analysis of these traits is described below.

### 2.4 Population trait differentiation between wild and captive environments

As a measure of among‐population plasticity in wild and captive environments, we compared female (and male) size‐at‐maturity, fecundity, egg size and GSI between captive‐born females in 2013 and wild females used to derive crosses in 2010, 2011 and 2014 (mean number of wild females/males per population = 38/52). Wild trait data were collected using the same procedures described as captive‐born females. Analyses of variance (ANOVAs) were employed to quantify the effect of population, environment (captive or wild) and the population × environment interaction for each trait except for male and female body mass (analysed using generalized linear models); between‐population comparison *p*‐values for each trait were independently adjusted to control for type‐I error using a Bonferroni correction. Trait differences between populations reared in contrasting environments were assessed using *lsmeans*.

### 2.5 Wild population survival in captivity

Wild population survival at different stages in the common hatchery environment was contrasted using generalized linear mixed‐effects models with a binomial error distribution: (i) egg to postyolk absorption across years (six populations); (ii) postyolk absorption to age 12 months across years (five populations); (iii) separate family rearing to age 7 months (eight populations, 2014); (iv) male mortality, observed at the onset and during the spawning period (eight populations, 2011); (v) egg to postyolk absorption for progeny of captive‐born adults (eight populations, November 2013). Population was a categorical fixed effect in all analyses, as was year in any models comparing survival over different cross years. Family and/or tank were included as random effects where applicable. Population‐level pairwise comparisons were conducted using *t* tests in *lsmeans*. An observation level random effect was fitted as needed to account for overdispersion (Browne, Subramanian, Jones & Goldstein, 2005).

Progeny of 2011 captive‐born adults was generated from gametes in October 2013 (mean families per population = 50, mean number of male/female parents per population = 22.5/20.6). Cross design and rearing conditions mirrored the parental generation except for a cooler incubation temperature that fluctuated naturally (1.8–4.7°C). We avoided inbred mating by tagging, genotyping and conducting parentage assignment of mature adults prior to crossing; with maximum 12 mothers per population and using 12 microsatellite loci, 100% parentage assignment was achieved (DNA extraction and amplification followed Bernos & Fraser, 2016; parentage assignment conducted in PAPA; Duchesne, Godbout & Bernatchez, 2002).

### 2.6 Correlates of plastic and genetic change among wild populations in captivity

We determined whether among wild populations in captivity, the extent of phenotypic changes, survival differences and lifetime success differences were negatively correlated with wild population attributes (using linear regressions in R, 2011 crosses). Wild population attributes considered were: (i) mean *N*_*b*_ (effective number of breeders), a strong analogue of contemporary *N*_*e*_ in Cape Race trout populations based on 5‐ to 7‐year time series of *N*_*b*_ data from Bernos and Fraser (2016); (ii) standing genetic variation (observed heterozygosity H_o_) from 164 neutral SNPs spread across chromosomes (Sauvage, Derome, Audet & Bernatchez, 2013; data from Fraser et al., 2014); and (iii) standing genetic variation from 12 microsatellite loci (H_o_ and allelic richness (*A*_*r*_); Bernos & Fraser, 2016). Data are summarized in Supporting Information Table S2. Lifetime success was defined as captive‐born survival probability within a population multiplied by captive‐born survival probability of the progeny produced by said population (to yolk absorption). Phenotypic changes were based on the degree of change in male/female body size at maturation and traits associated with female reproductive investment between the wild and captivity.

Finally, to investigate the potential release of genetic variation in captivity for each trait assayed, we tested whether captive‐born population trait variability (coefficient of variation (CV)) was positively correlated with wild population *N*_*b*_.

## 3 RESULTS

### 3.1 Wild population trait differentiation in captivity

**Figure 1:**
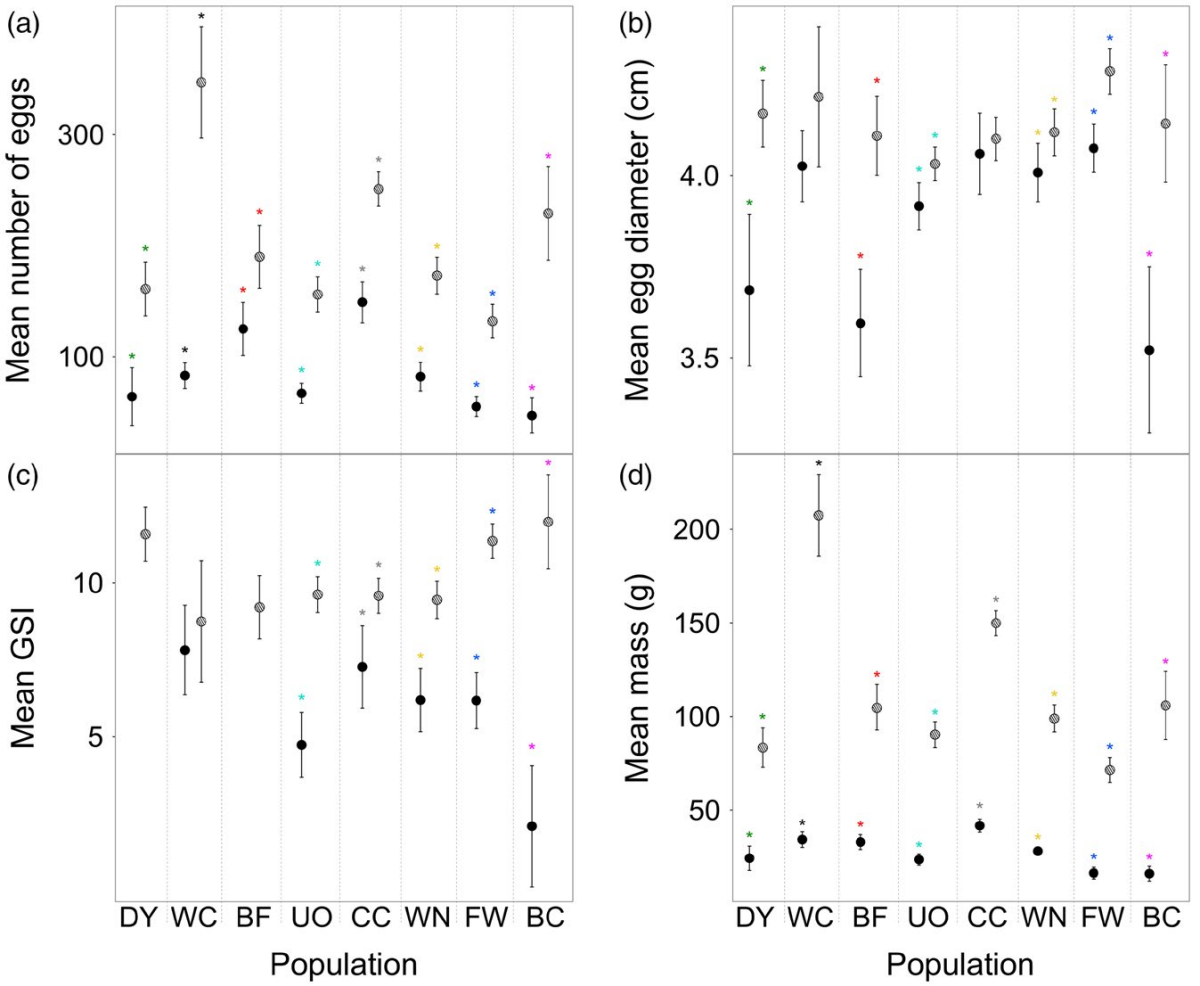
Female trait data for wild (filled circle) versus captive‐born (hatched circle) brook trout from different Cape Race populations: (a) mean number of eggs; (b) mean egg diameter; (c) mean gonadosomatic index (GSI); (d) mean mass at the spawning period. Bars are 95% confidence intervals. Populations are shown in increasing order of their effective number of breeders (*N*_*b*_) in the wild. A star represents a significant pairwise comparison between wild versus captive‐born traits. Based on 2011 crosses

Populations had similar length–mass relationships in captivity (Supporting Information Figure S3), but growth rates differed significantly (Table 1, Supporting Information Table S3; pairwise comparisons in Supporting Information Table S4). Body size increasingly diverged with time among populations over the first year (Tables 1 and 2; pairwise comparisons in Supporting Information Tables S5, S6). By 18 months, mean mass varied 2.1‐fold for males and 2.9‐fold for females: CC and WC were significantly larger, FW was significantly smaller, and all other populations were intermediate in body size (Supporting Information Figure S3, Tables 2 and 3). Populations with close genetic relationships (WN‐BF, UO‐DY) had similar body sizes over most or all time periods (Figure 1, Supporting Information Figure S3; Table 2, Supporting Information Tables S4–S8).

**Table 1:** Statistical summary of model selection for captive‐born Cape Race brook trout length and mass (from 0 to 12 months, using Likelihood ratio tests), captive‐born survival across different life stages or when reared as separate families, and progeny survival of captive‐born adults (parents from 2011 crosses)

Model no.	Description	Versus model no.	Log‐likelihood	Term	*Χ* ^2^	*df*	*p*
Length							
0^a^	P + T + P:T	‐	−10,779.0	‐	*‐*	*‐*	*‐*
1	P + T	0	−10,809.7	P:T	61.4	6	<0.001
Mass							
0^a^	P + T + P:T	‐	−719.5	‐	‐	‐	‐
1	P + T	0	−747.4	P:T	55.8	6	<0.001
Survival from egg to yolk absorption (2011+2014)							
0^a^	P + Y + P:Y	‐	−1,425.7	‐	*‐*	*‐*	*‐*
1	P + Y	0	−1,431.3	P:Y	11.163	5	0.048
Survival from yolk absorption to one year (2011+2014)							
0	P + Y + P:Y	‐	−61.9	‐	‐	‐	‐
1	P + Y	0	−64.7	P:Y	5.631	4	0.229
3^a^	P	1	−66.0	Y	2.650	1	0.104
4	Intercept only	2	−102.4	P	72.721	4	<0.001
Survival to 7 months (2014 only, family‐level random effect)							
0^a^	P		−124.6				
1	Intercept only	0	−160.9	P	72.611	7	<0.001
Survival of progeny generated from captive‐born adults							
0^a^	P		−1,041.2				
1	Intercept only	0	−1,066.3	P	50.24	7	<0.001

*Notes*.

P: population; Y: year; T: time period.

a Selected model.

**Table 2:** Mean body size (length in mm and mass in g), ±1 standard error of the mean (parentheses) of captive‐born Cape Race brook trout populations in a common hatchery environment at age 0 (yolk absorption), 3, 7, 12 and 18 months (maturation). Based on 2011 crosses

Population	Age 0	3 months		7 months		12 months	
	Length	Length	Mass	Length	Mass	Length	Mass
FW	22.83 (0.08)	47.06 (0.36)	1.09 (0.03)	68.69 (0.74)	4.24 (0.15)	101.9 (0.97)	11.6 (0.45)
BC	20.41 (0.13)	50.47 (0.64)	1.22 (0.05)	79.25 (1.40)	6.93 (0.29)	119.1 (2.77)	22.9 (1.28)
DY	23.68 (0.11)	47.96 (0.45)	1.02 (0.03)	73.76 (0.81)	5.17 (0.17)	111.5 (1.21)	14.6 (0.56)
UO	23.64 (0.07)	50.38 (0.36)	1.37 (0.03)	76.71 (0.74)	5.23 (0.15)	112.4 (0.99)	14.6 (0.46)
BF	23.25 (0.11)	48.66 (0.36)	1.20 (0.03)	74.54 (0.74)	5.20 (0.15)	113.4 (1.18)	16.1 (0.55)
WN	23.85 (0.10)	49.90 (0.36)	1.24 (0.03)	75.79 (0.74)	5.16 (0.13)	113.7 (0.99)	16.5 (0.46)
WC	23.41 (0.08)	51.25 (0.36)	1.35 (0.03)	84.33 (0.77)	7.00 (0.16)	135.0 (1.27)	25.8 (0.58)
CC	24.54 (0.08)	52.60 (0.37)	1.38 (0.03)	80.59 (0.74)	5.80 (0.15)	129.2 (0.99)	22.1 (0.46)

Population	18 months, ♂		18 months, ♀	
	Length	Mass	Length	Mass
FW	185.7 (2.4)	89.1 (3.8)	179.7 (2.5)	71.4 (4.0)
BC	206.7 (9.0)	118.5 (14.3)	199.0 (6.9)	105.9 (11.1)
DY	206.2 (2.6)	122.6 (4.1)	190.9 (4.0)	83.4 (6.4)
UO	215.1 (2.4)	132.5 (3.7)	197.3 (2.6)	90.3 (4.2)
BF	215.5 (3.6)	137.8 (5.8)	203.6 (4.7)	105.0 (7.5)
WN	213.9 (2.3)	132.0 (3.7)	203.9 (2.8)	98.9 (4.4)
WC	251.4 (3.7)	187.7 (5.9)	259.6 (8.3)	207.5 (13.3)
CC	241.2 (2.5)	166.1 (3.9)	232.6 (2.6)	149.9 (4.1)

**Table 3:** Results of model selection for length and mass of captive‐born Cape Race brook trout populations at 18 months in a common hatchery environment, using *F* tests

Parameter	Length		Mass		
	*F* value	*p* value	*F* value	*df*	*p* value
Population:Sex	1.43^7^_833_	0.189	3.68^7^_833_	7	<0.001
Population	116.78^7^_840_	<0.001	121.44^7^_833_	7	<0.001
Sex	46.89^1^_840_	<0.001	99.17^1^_833_	1	<0.001

Population, sex and their interaction all had significant effects on body size at 18 months, the latter because captive‐born males were significantly larger than females in six of eight populations but the same size in two populations (Supporting Information Figure S3, Table 4). Among captive‐born populations, male and female body size at maturation was largely consistent with body sizes at age 12 months (Table 2; pairwise comparisons in Supporting Information Table S8).

**Table 4:** Results of model selection for traits associated with female reproductive investment, female size (mass) and male size (mass) in Cape Race brook trout populations between the wild and when reared in a common hatchery environment

Parameter	Fecundity		Egg diameter		GSI	
	*F* value	*p* value	*F* value	*p* value	*F* value	*p* value
Population:Environment	7.1^7^_668_	<0.001	5.4^7^_555_	<0.001	8.1^5^_434_	<0.001
Population	56.9 ^7^_668_	<0.001	8.2^7^_555_	<0.001	8.0^7^_434_	<0.001
Environment	474.1^1^_668_	<0.001	63.5^1^_555_	<0.001	213.2^1^_434_	<0.001

Parameter	Female mass		Male mass	
	*F* value	*p* value	*F* value	*p* value
Population:Environment	27.5^7^_716_	<0.001	14.0^7^_910_	<0.001
Population	80.7^7^_716_	<0.001	60.1^7^_910_	<0.001
Environment	1,787.7^1^_716_	<0.001	2,863.4^1^_910_	<0.001

Mean captive‐born female fecundity, egg size and GSI varied 2.6‐fold, 1.1‐fold and 1.4‐fold, respectively, among populations at maturation (Figure 1a–c; Table 4, Supporting Information Tables S9–S11). Larger body‐sized populations had higher fecundity (e.g., WC, CC) but not necessarily larger egg size (see FW; Figure 1b/d).

### 3.2 Population trait differentiation between wild and captive environments

Captive‐born individuals of both sexes were significantly larger than their wild counterparts at maturation (Figure 1d; Table 4; male data in Supporting Information Figure S4). Captive‐born females also had larger or similar trait values associated with reproductive investment (Figure 1a–c; Table 4).

We detected population differences in plasticity, evidenced by a significant population‐by‐rearing environment interaction in all traits assayed between captive and wild environments (Table 4). Per trait, more variation was explained by rearing environment, followed by population, and then by the population × rearing environment interaction (Table 4). The absolute difference in female fecundity between captive and wild environments was greatest in the largest body‐sized populations (WC, CC, BC) and least in BF (Figure 1a). For egg size and GSI, three populations had disproportionate mean trait increases from the wild to captivity (respectively DY, BF, BC and UO, FW, BC; Figure 1b,c).

### 3.3 Wild population survival in captivity

**Figure 2:**
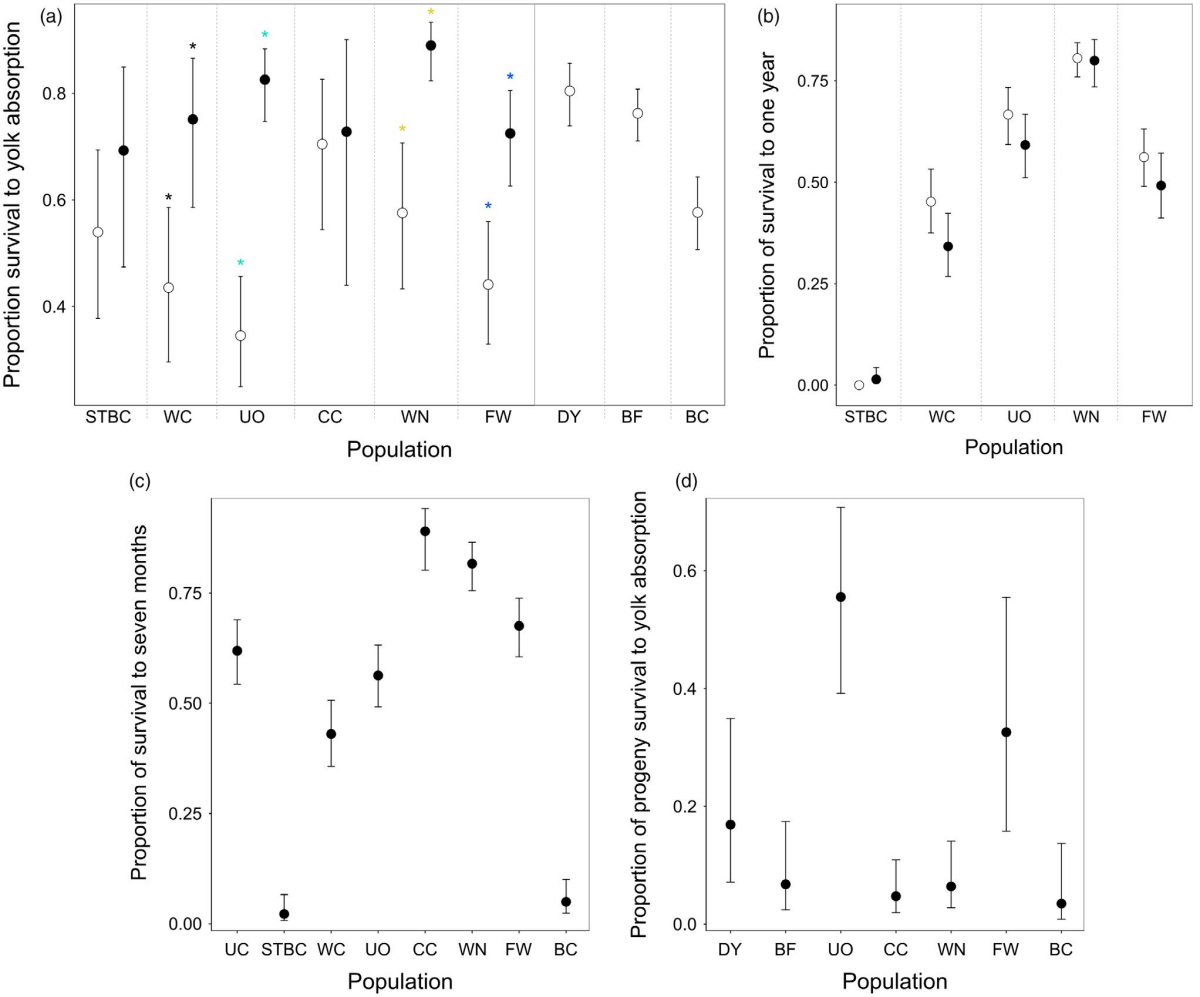
Proportion of survival to (a) yolk absorption and to (b) one year for captive‐born Cape Race brook trout populations reared in a common hatchery environment (2011, open circles; 2014, filled circles). Bars are 95% confidence intervals. A star represents a significant pairwise comparison within a population. Populations are in increasing order of effective number of breeders (*N*_*b*_) in the wild. Pairwise population comparisons for (b) are found in Supporting Information Table S11; pairwise population comparisons for (a) are not reported, as only FW‐WN were statistically different across experimental years. (c) The proportion of survival when captive‐born brook trout were reared as separate families to seven months (based on 2014 crosses), in a common hatchery environment. (d) The proportion of survival to yolk absorption of progeny generated from captive‐born adults (based on 2013 crosses), in a common hatchery environment

Mean captive‐born survival differed several‐fold among wild populations at each life stage (Figure 2; Table 1). Experimental year effects were observed from egg to postyolk absorption (Figure 2a), but population survival differences were greatest and highly consistent across years from postyolk absorption to age 12 months (Figure 2b; Supporting Information Table S12). Ranking of populations was similar when families were reared separately versus when pooled (Figure 2a–c: WN, CC higher survival; UO, FW intermediate survival; WC low survival; STBC, BC very low survival). Less studied DY and BF had either lower or similar survival relative to their closely related populations (UO, WN, respectively; Figure 2).

**Figure 3:**
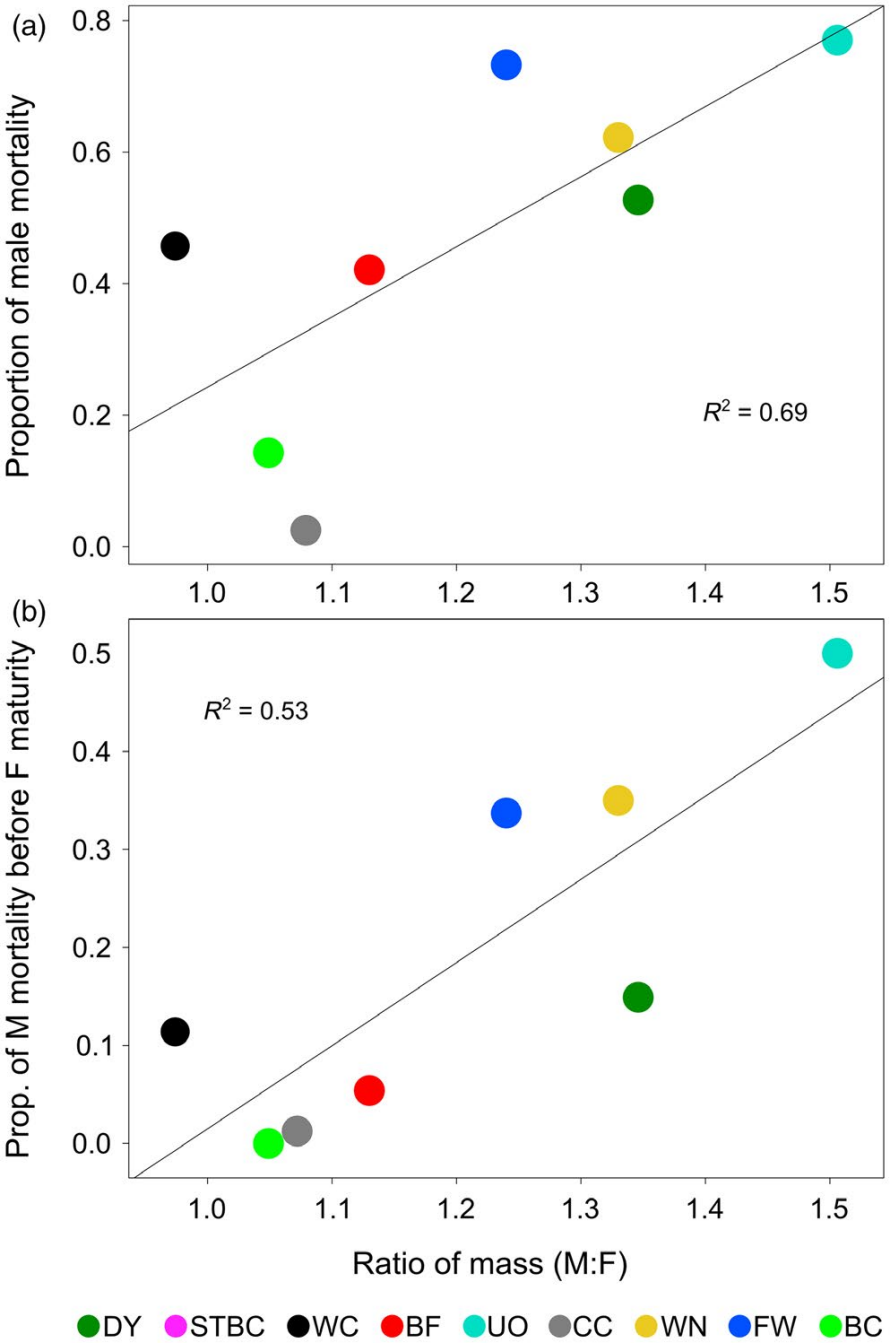
The relationship between the difference in body size between captive‐born males versus females within a Cape Race trout population when reared in a common hatchery environment and (a) total male mortality over the reproductive period in captivity or (b) male mortality before females began maturing in captivity (based on 2011 crosses)

Extensive male mortality occurred at the beginning and throughout the reproductive period and terminated thereafter (Supporting Information Figure S5; female mortality during this time was negligible); all dead males produced sperm, but were never used to generate crosses. Populations with disproportionately faster‐growing males than females experienced higher male mortality. This occurred both before any females had ripe eggs within the respective population (linear *R* ^2 ^= 0.69, *p *<* *0.01) and cumulatively over the entire reproductive period (linear *R* ^2 ^= 0.53, *p *=* *0.03) (Figure 3).

**Figure 4:**
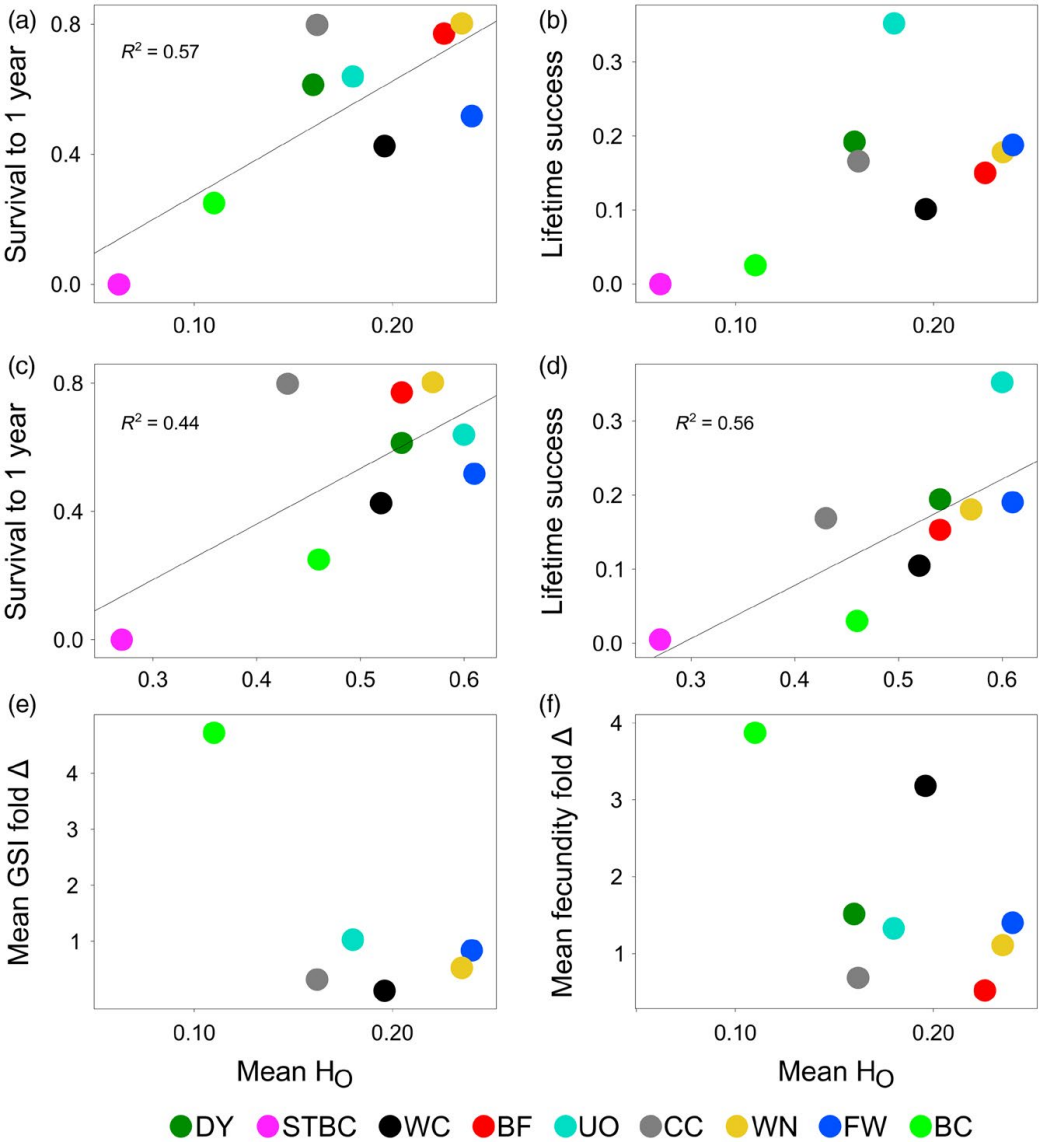
The relationship between the heterozygosity (H_o_) in a wild population of Cape Race brook trout using SNPs and (a) mean survival to one year in captivity or (b) lifetime success in captivity (i.e., lifetime survival and reproductive success) (based on 2011 crosses). (c) and (d) are the same relationships as (a) and (b) respectively but using wild population H_o_ based on microsatellites. The relationship between wild population H_o_ using SNPs and the extent of phenotypic change experienced in captivity for (e) female gonadosomatic index (GSI), and (f) female fecundity. See Supporting Information Figures S7–S9 for remaining relationships between various genetic variables in the wild populations of Cape Race brook trout and their survival or phenotypic change in captivity. Figure panels (b, e and f) do not show regression lines as none of their relationships were statistically significant; they are nonetheless included to help visualize trends described in the main text, and to facilitate comparisons between results when SNPs or microsatellites were adopted

Progeny survival of captive‐born adults had a 6.6‐fold range of variation among populations; notably, UO progeny had higher survival than all other populations (Figure 2d; pairwise contrasts not shown). No relationship existed between survival of captive‐born adults and survival of their progeny (Supporting Information Figure S6). All populations produced some captive‐born females having poor‐quality eggs (determined when none developed to the eyed stage), but the proportion varied considerably among populations (Supporting Information Table S13). Together, these survival differences translated into a 14‐fold range of variation among populations in lifetime success within a common hatchery environment, even after excluding STBC which had a lifetime success of zero (Figure 4b,d).

### 3.4 Correlates of plastic and genetic change among wild populations in captivity

Compared to populations with higher H_o_, populations with lower H_o_ experienced higher mortality in captivity (SNPs: *R* ^2 ^= 0.57, *p *=* *0.02; microsatellites: *R* ^2 ^= 0.44, *p = *0.05; Figure 4). Populations with lower H_o_ also had or tended to have lower lifetime captive success (SNPs: *R* ^2 ^= 0.34, *p *=* *0.10; microsatellites: *R* ^2 ^= 0.56, *p = *0.02) (Figure 4). At two of five traits, higher mortality in lower H_o_ populations translated into trends for their survivors to deviate more from wild phenotypic expression than survivors from wild populations in which captive mortality was low (female GSI: *R* ^2 ^= −0.54, *p = *0.09; female fecundity, *R* ^2 ^= −0.32, *p = *0.14) (Figure 4, Supporting Information Figure S7). Only one significant relationship was detected between a wild population's *N*_*b*_ or *A*_*r*_ and five different metrics of change in captivity (the change in male mass increased as *N*_*b*_ increased; *R* ^2 ^= 0.59, *p = *0.02) (Supporting Information Figures S8 and S9). Only one of five captive population trait CVs was positively correlated with wild population *N*_*b*_ (female fecundity; *R* ^2 ^= 0.60, *p = *0.01); no relationships were detected at the other four traits (Supporting Information Table S14).

## 4 DISCUSSION

A single generation of captive exposure induced a wide range of plastic/genetic changes among nine brook trout populations at different life stages and between sexes. Wild populations with lower heterozygosity experienced the greatest mortality in captivity and generally had lower lifetime success in captivity; for two of five traits, their survivors also tended to deviate most in trait expression relative to their wild counterparts. Greater male‐biased mortality in captivity occurred in populations where males had disproportionately higher growth rates than females. Survival differences among populations under common, captive environmental conditions were repeatedly demonstrated across independent experimental years, irrespective of whether families were pooled within populations or reared separately. Causal mechanisms driving differential wild population performance in captivity were not investigated but are undoubtedly influenced by numerous factors. Based on observations during rearing and links to other studies below, we speculate these factors could include altered population‐dependent growth costs of reproduction between the sexes, differential water‐borne pathogen or bacterial resistance and stress tolerance.

Our metric of single generation, lifetime success in captivity (with an average value of 0.15, range 0–0.35; Figure 4) combined the probability of surviving to maturation for captive‐born fish with the early survival probability of their progeny. This metric is not comparable to previous studies on other salmonids that contrasted reproductive success of wild and captive‐born adults in the wild (Araki et al., 2007; Christie et al., 2012; Milot, Perrier, Papillon, Dodson & Bernatchez, 2013). At a minimum, low success and the plastic/genetic changes observed in a single generation of captivity for brook trout are consistent with theory and with previous empirical studies demonstrating a high potential for the captive environment to generate rapid maladaptation in wild species (Araki et al., 2007; Christie, Marine, Fox, French & Blouin, 2016; Christie et al., 2012; Frankham, 2008; Fraser, 2008; Milot et al., 2013). Cape Race trout populations also have no history of human influence and long evolutionary histories in isolation (Danzmann et al., 1998; Wood et al., 2014). We suspect that the extent of Cape Race trout differentiation might resemble that expected among wild populations being used in larger scale conservation programs for species with similar fecundities (e.g., some other fishes, amphibians, insects) and/or with a similar timeframe for population differentiation to arise (e.g., species occupying de‐glaciated regions). A similar extent of plastic and genetic change among populations in captivity might not arise if differences between captive and wild environments are thoroughly minimized, including growth differences (e.g., Berejikian et al., 2012; Campbell, Beckman, Fairgrieve, Dickey & Swanson, 2006; Fraser, 2008; Larsen et al., 2006). Yet, many characteristics of our captive conditions were common to those of captive/supplementation programs for salmonids (e.g., rearing densities, aquaculture feed, seasonally fluctuating temperatures, rearing tanks without substrate/shelter, no predators: Jonsson & Jonsson, 2006; Araki et al., 2007; Fraser, 2008; Clarke, Fraser & Purchase, 2016).

Long‐term population sizes of Cape Race trout populations are unknown. Contemporary adult census population sizes and *N*_*b*_ fluctuate interannually. However, the magnitude of among‐population differences has been consistent for seven years (Bernos & Fraser, 2016) and is strongly correlated with stream drainage sizes which have been stable for much longer (Wood et al., 2014). In models of isolated populations, long‐term *N*_*e*_ and H_o_ should be positively correlated (Frankham, Briscoe & Ballou, 2002). Yet only H_o_ and not contemporary *N*_*b*_ was correlated with the extent of phenotypic change experienced by wild Cape Race populations in captivity. H_o_ within populations may better reflect long‐term *N*_*e*_ than contemporary *N*_*b*_. Certain populations with lower H_o_ (e.g., BC, STBC) may have also experienced short‐term historical bottlenecks not detected in previous works to explain their poor performance when transplanted to captivity. Furthermore, eastern Cape Race populations (WN, BF, CC) survived well to maturation in captivity but had proportionally more females with poorer egg quality and hence poorer captive‐born progeny survival (Supporting Information Figure S6). Together, these results point to the importance of fine‐scale evolutionary history of populations in influencing their responses to contemporary environmental changes.

Larger wild populations only exhibited more trait variability in captivity than smaller populations (despite similar family representation) for one of five traits assayed (female fecundity). Such differential plastic expression of presumably neutral genetic diversity might have contributed to the differential performance of Cape Race populations in captivity. However, the lack of correlation between *N*_*b*_ and lifetime success suggests that the conversion of plastic trait expression into adaptation to novel environmental change is not a straightforward process—or that the types of traits we assayed are not strong predictors of this process.

Males commonly grew larger than females in captivity. The greater the sex difference within a population, the more male mortality was incurred just before or during the female reproductive period. Male Cape Race trout invest more energy into reproduction than females, resulting in a greater cost of reproduction, commonly manifesting as greater postreproductive mortality (Hutchings, 2006; Hutchings, Pickle, McGregor‐Shaw & Poirier, 1999). Hutchings (2006) did not detect an influence of growth rate on trout survival, but admitted such an effect was difficult to detect in nature given the slow growth rate of Cape Race trout. Captive growth rate was considerably faster than in the wild, as is common in many fish hatchery environments (e.g., Araki et al., 2007; Berejikian et al., 2012; Campbell et al., 2006; Fraser, 2008; Larsen et al., 2006). Observed male mortality in captivity could relate to a substantial survival consequence to placing so much energy into reproduction; population differences in these costs may relate to differences in opportunities for postmaturational growth increase (Hutchings, 2006). Moreover, populations varied in the proportion of captive females producing poor‐quality eggs. There are two sex‐specific conservation implications here. For males, resulting mortality from altered growth rates in captivity (and presumably their associated physiological consequences, e.g., see Larsen et al., 2006) may shift locally adapted life history reaction norms for growth, survival and reproduction. For females, we suspect that captivity induces population‐dependent levels of stress which affect maternal investment and offspring survival.

Our study also contributes to a growing literature in detecting remarkable, genetically based population differentiation at a fine scale (populations are separated 0.3–10 km) (e.g., Richardson, Urban, Bolnick & Skelly, 2014). Cape Race population differentiation is certainly facilitated by long‐term physical isolation and different local and seasonal environmental features within streams, which can lead to adaptive differentiation (Fraser et al., 2014; Hutchings, 1993, 1996; Wood et al., 2015; Zastavniouk et al., 2017).

### 4.1 Conservation and evolutionary implications

Our research suggests that the scope of maladaptive effects to wild fitness from single generation captive exposure could vary considerably among populations within a given species and between the sexes. This should be factored in whenever a decision is being made on whether, and how, to initiate a species conservation program involving captive breeding or rearing. It also suggests that quantitative modelling, if based on data from one or a few populations or only one sex (e.g., Bowlby & Gibson, 2011), will likely not capture the full breadth of the influence of maladaptation from captive exposure on wild population recovery within a species. While our results are compelling because of the scope of deviations from wild phenotypes and fitness, we cannot be certain that the trait expression observed in captivity would necessarily be maladaptive in the wild.

We also find that some commonly adopted metrics of past and current population genetic monitoring efforts (contemporary *N*_*b*_ and *A*_*r*_) appear to be poor predictors of short‐term population responses to novel environmental change (in this case, captive conditions). Conversely, heterozygosity appears to be a better candidate as a predictor and one that might more closely reflect long‐term population size dynamics; it is also an increasingly simple metric to assay genomewide in wild populations (e.g., Allendorf, Hohlenhohe & Luikart, 2010).

Our study additionally provides a rare empirical test on vertebrates of the role that genetic diversity plays in responses to environmental change. In so doing, we have illustrated a conservation conundrum: some of the populations of most conservation concern (e.g., those with low heterozygosity) potentially become the most maladapted from being in captivity, at least in short‐term (single generation) captive rearing programs. Our research on Cape Race trout suggests that viability selection, including through maternal effect influences, is an especially important primer of maladaptation from captive exposure. The generality of these research findings should be tested/evaluated in other species and population systems.

Because our results suggest that the risk of maladaptively changing low heterozygosity populations in captivity might be high, further research should experimentally test the benefits of facilitating gene flow from ecologically similar wild populations (i.e., genetic‐rescue) at the initiation of captive rearing.

Common garden research studies such as ours shed light on the adaptive potential of wild populations. The extent of environmental change going from the wild to captive environment was clearly too much for some Cape Race trout populations with low heterozygosity. Encouragingly, however, in the wild these populations have persisted in isolation for long periods despite low genetic diversity, and hence appear quite adept at handling natural rates of environmental change.

## CONFLICT OF INTEREST

None declared.

## DATA ARCHIVING STATEMENT

Data available from the Dryad Digital Repository: https://doi.org/10.5061/dryad.m6383tr
