# Supplementary material for: Population correlates of rapid captive‐induced maladaptation in a wild fish
Source: Evol Appl. 2018 Jun 19;12(7):1305–17. doi: 10.1111/eva.12649 (PMC6691219; doi:10.1111/eva.12649)
Supplement: Supplementary file 1 [file EVA-12-1305-s001.doc]

Appendix. Supplementary results and information for “Population correlates of rapid captive-induced maladaptation in a wild fish”


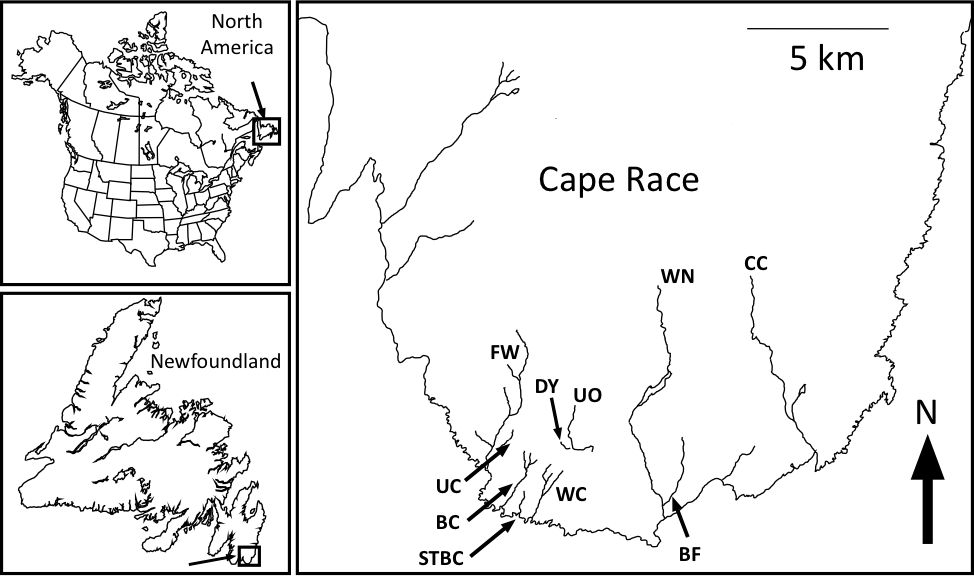


Figure A1. Map of the study’s wild brook trout populations from Cape Race, Newfoundland, Canada. Population abbreviations (from west to east): FW = Freshwater River; UC = Upper Coquita stream; BC = Bob’s Cove River; STBC = Still There By Chance; WC = Whale Cove River; DY = Ditchy Brook; UO = Upper Ouananiche Beck; WN = Watern Cove River; BF = Blackfly River; CC = Cripple Cove River


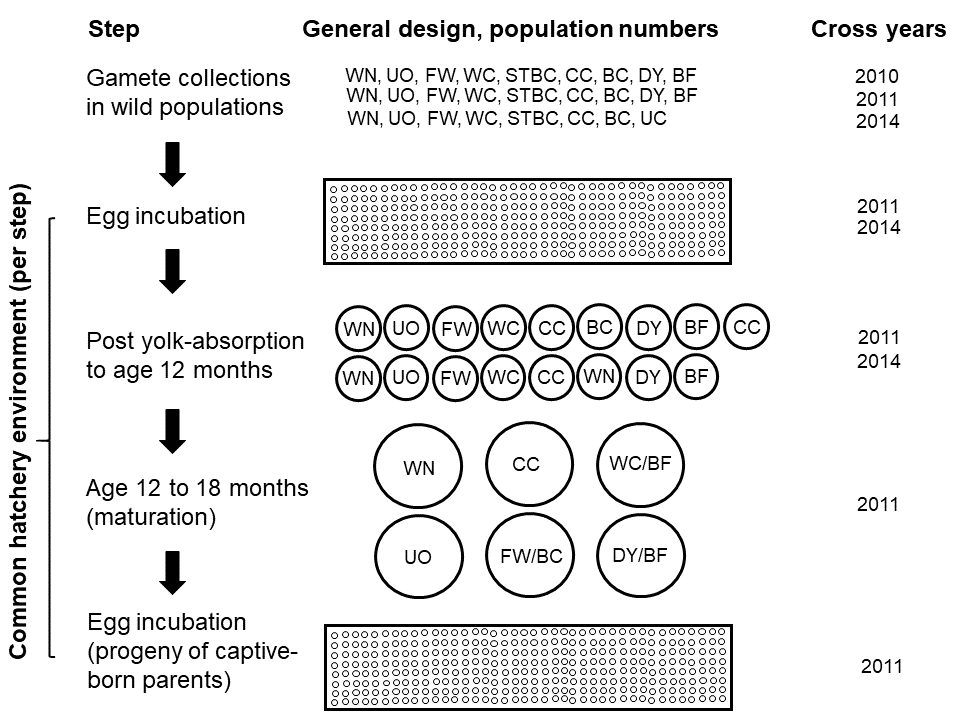


Figure A2. Schematic design of the general experimental set up for the rearing of captive-born brook trout from different wild populations of Cape Race, in a common hatchery environment. Post-yolk absorption to age 12 months is visualized for 2011 crosses only. Not shown is the separate family rearing conducted from post-yolk absorption to age 7 months based on 2014 crosses. Details are found in the main text.


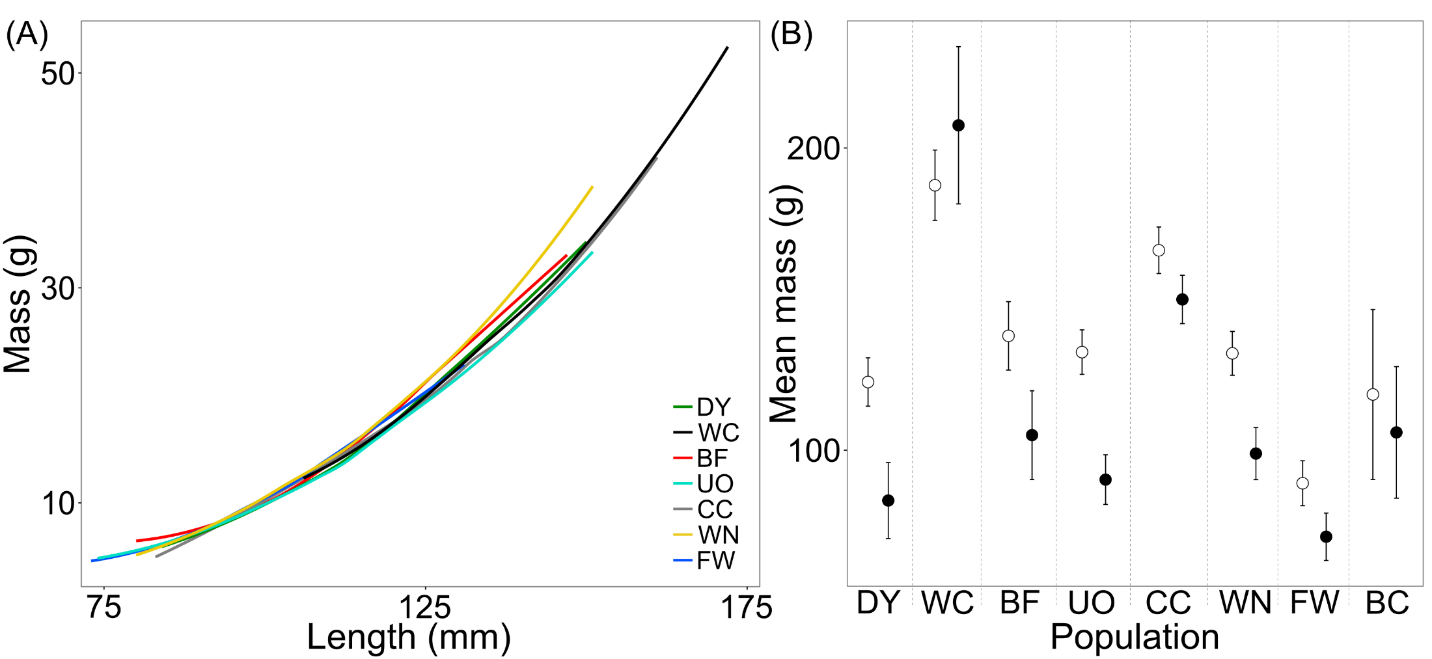


Figure A3: (A) Length-mass curves at 12 months of age for captive-born Cape Race brook trout populations reared in a common hatchery environment; (B) Mean mass for captive-born males (open circles) and captive-born females (filled circles) at 18 months of age among populations in a common hatchery environment, shown in increasing order of effective number of breeders (*N_b_*) in the wild populations. Bars in (B) are 95% confidence intervals.


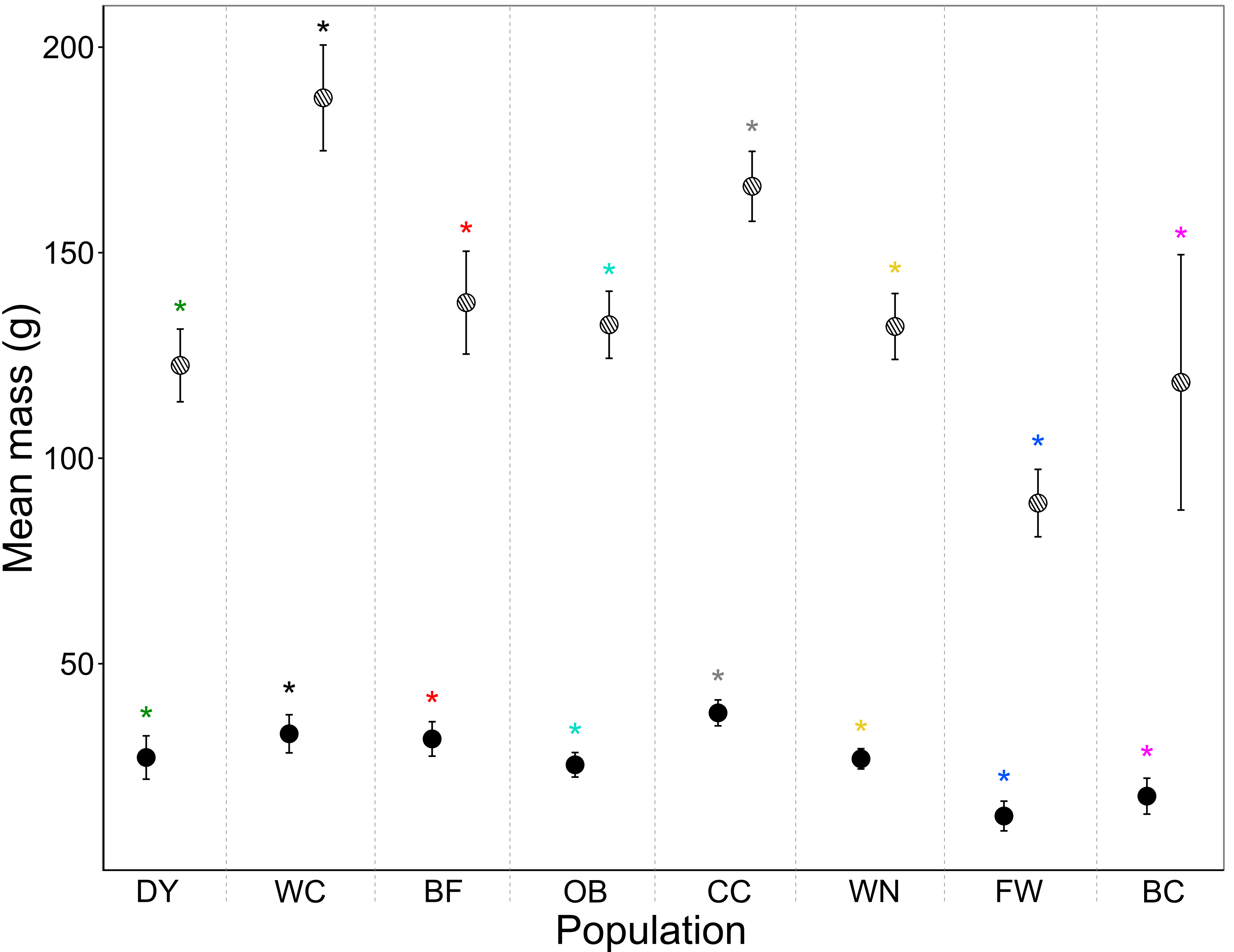


Figure A4. Least squares mean mass for wild males (filled circle) vs. captive-born males (hatched circle) at the spawning period. Bars are 95% confidence intervals. Cape Race brook trout populations are shown in increasing order of effective number of breeders (*N_b_*) in the wild. A star represents a significant (after Bonferroni correction) pairwise comparison between wild and captive-born. Based on 2011 crosses.


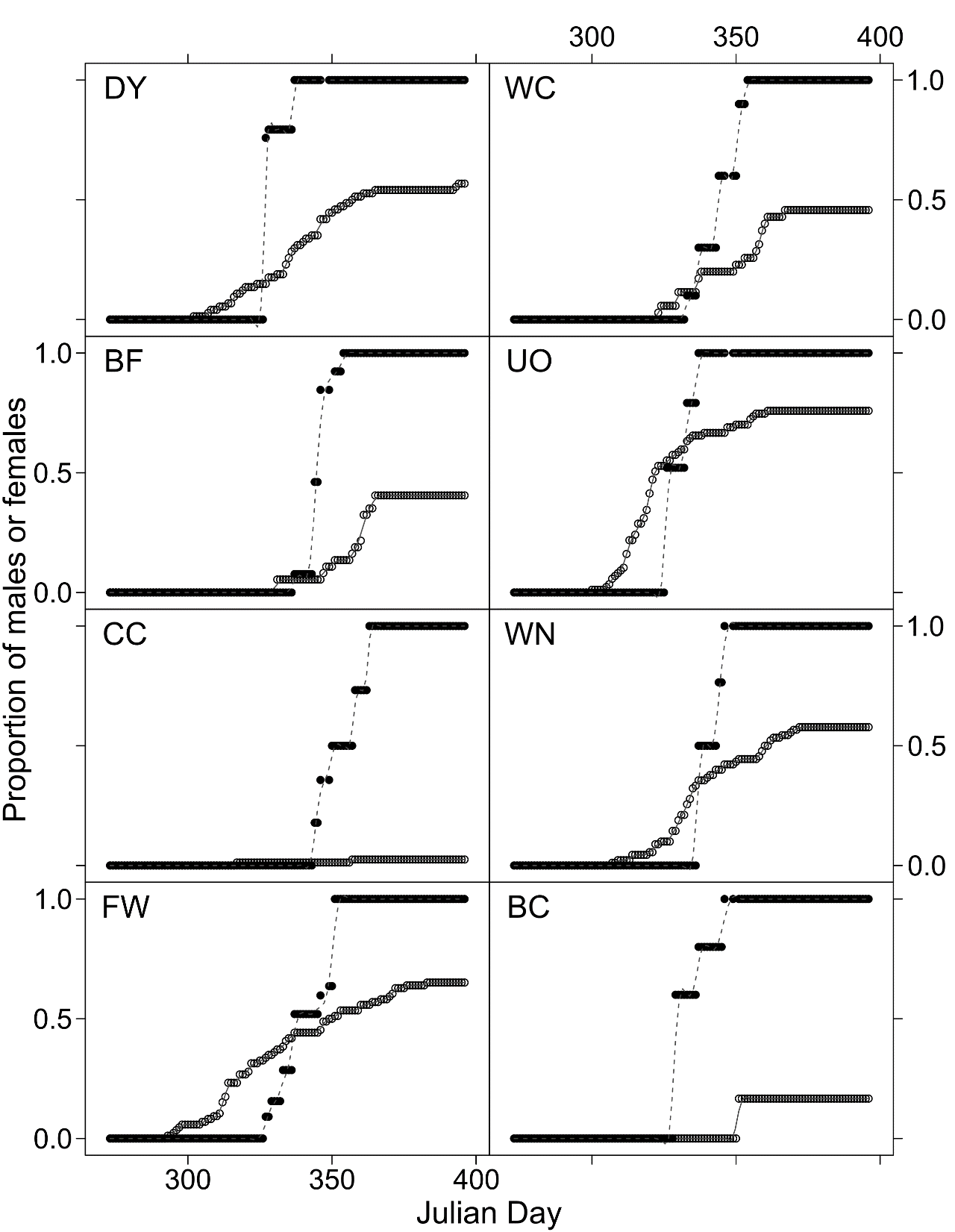


Figure A5. Proportion of spawning, captive-born females within each population of Cape Race brook trout on a given Julian day (filled circles, dashed line) in a common hatchery environment, relative to cumulative male mortality (open circles, solid line). Based on 2011 crosses.


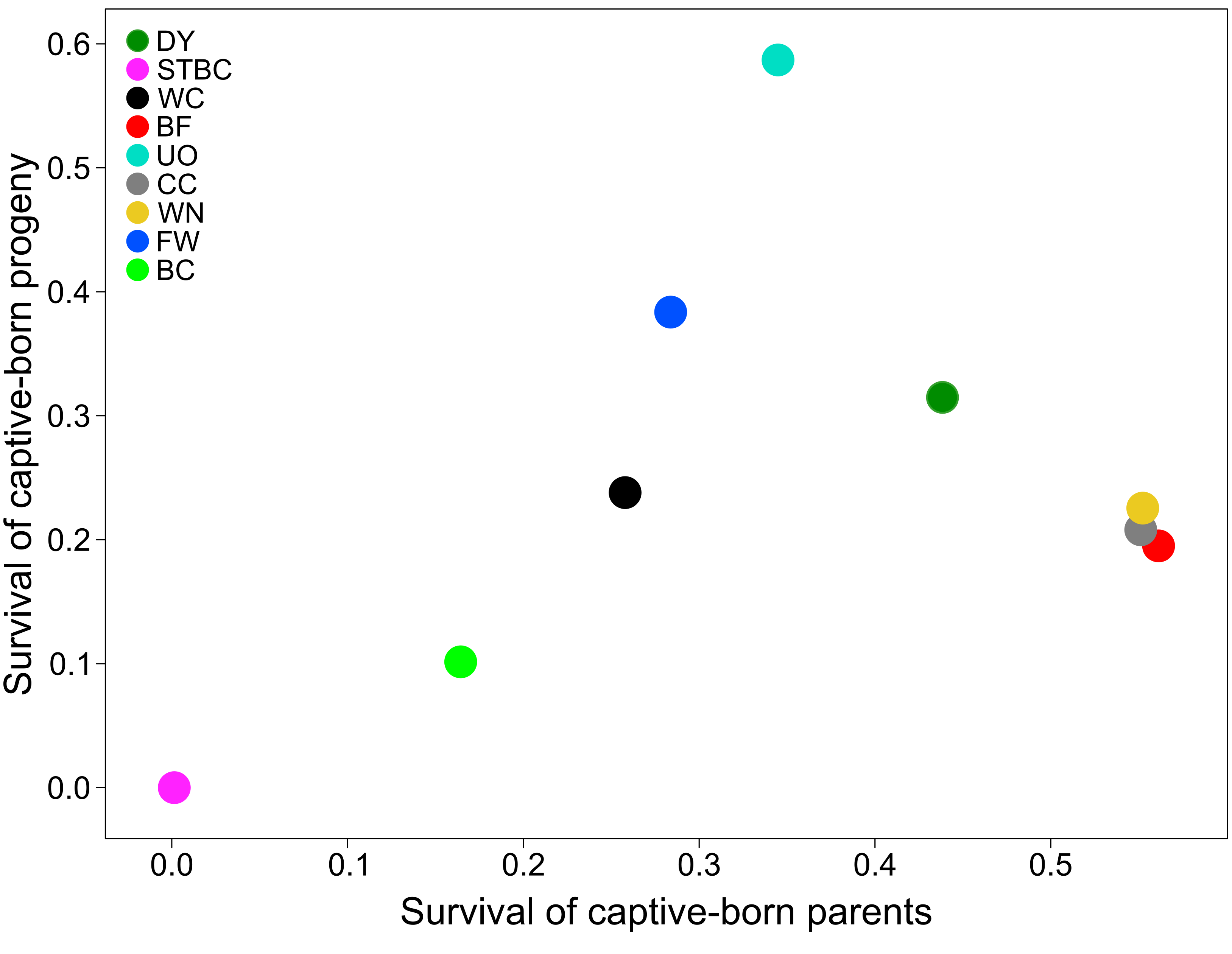


Figure A6. Relationship between survival of captive-born parents among Cape Race brook trout populations in a common hatchery environment (based on 2011 crosses), and the survival of their captive-born progeny (also reared in a common hatchery environment).


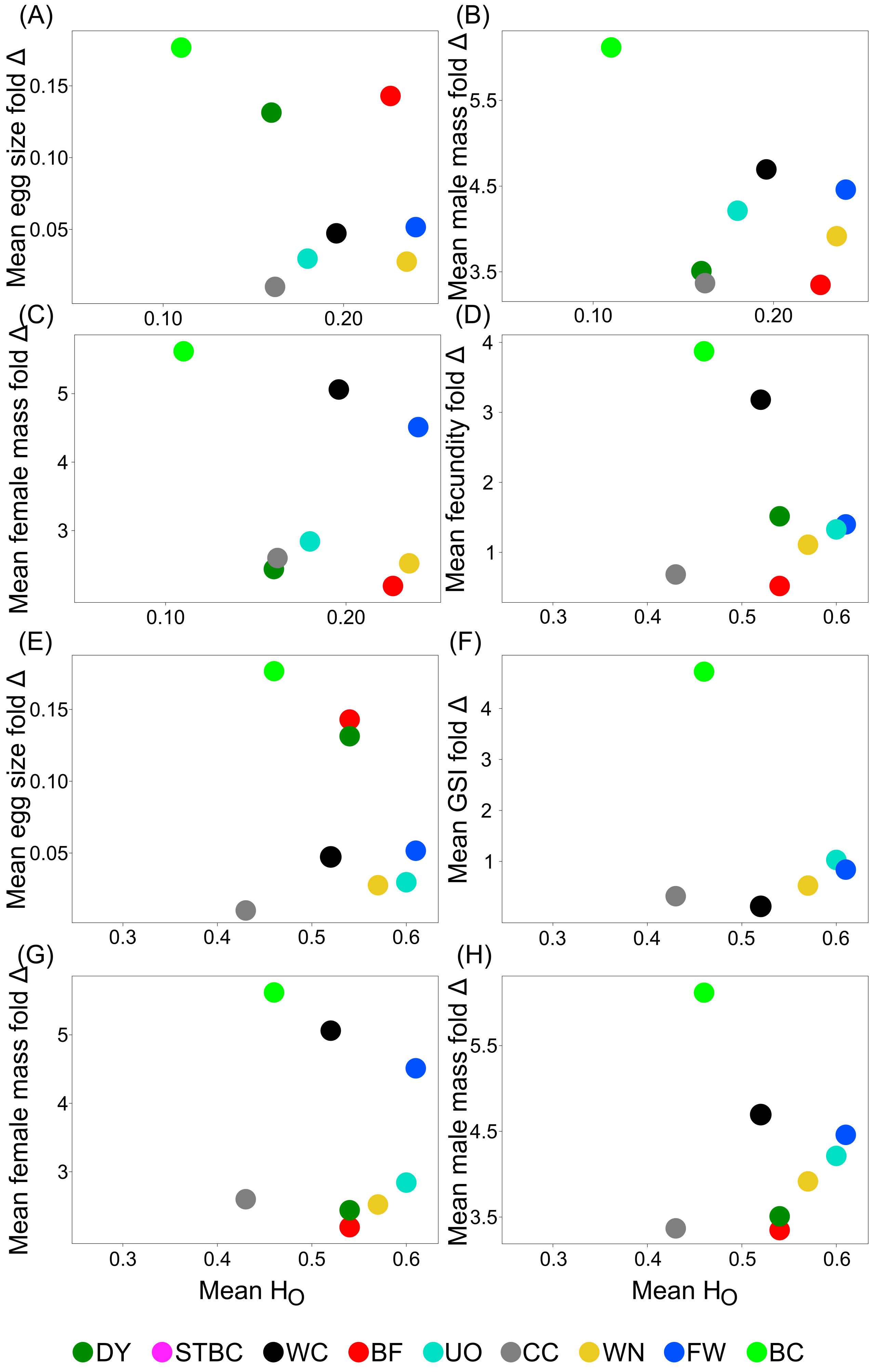


Figure A7. Relationship between the SNP heterozygosity (H_o_) within a wild population of Cape Race brook trout and the extent of phenotypic change observed in captivity at different traits (A-C); panels D-H show the same comparisons using microsatellite H_o_. Based on 2011 crosses.


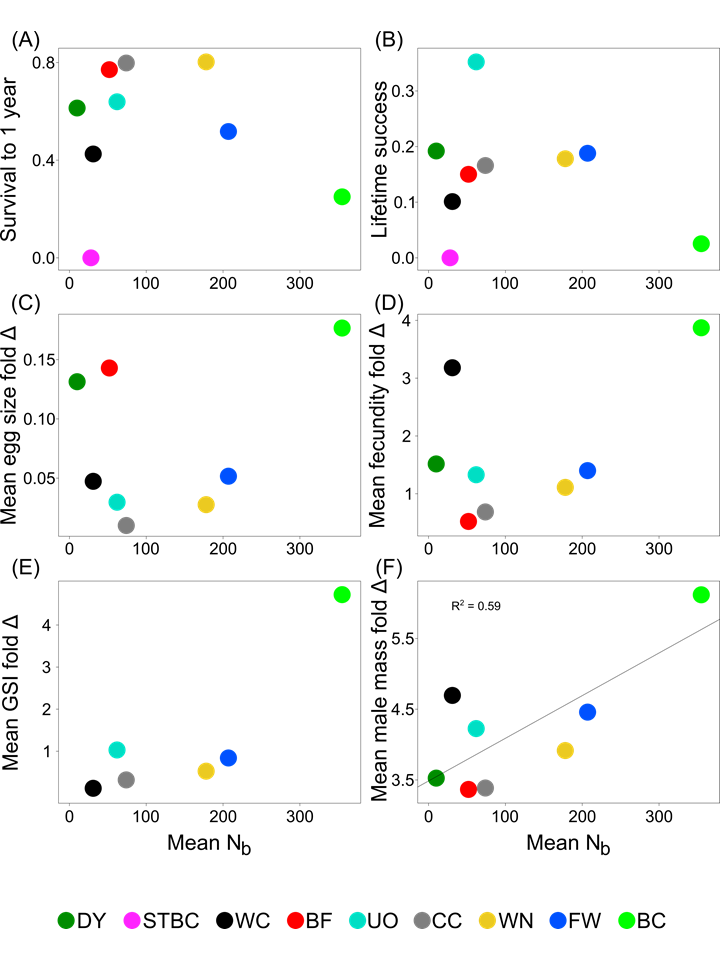


Figure A8. Relationship between the effective number of breeders (*N_b_*) of different wild populations of Cape Race brook trout using microsatellite loci and (A) mean captive-born survival to one year, (B) lifetime success in captivity, and the extent of phenotypic change experienced in captivity at different traits (C-F). Based on 2011 crosses. Regression lines are only included where the relationship was statistically significant.


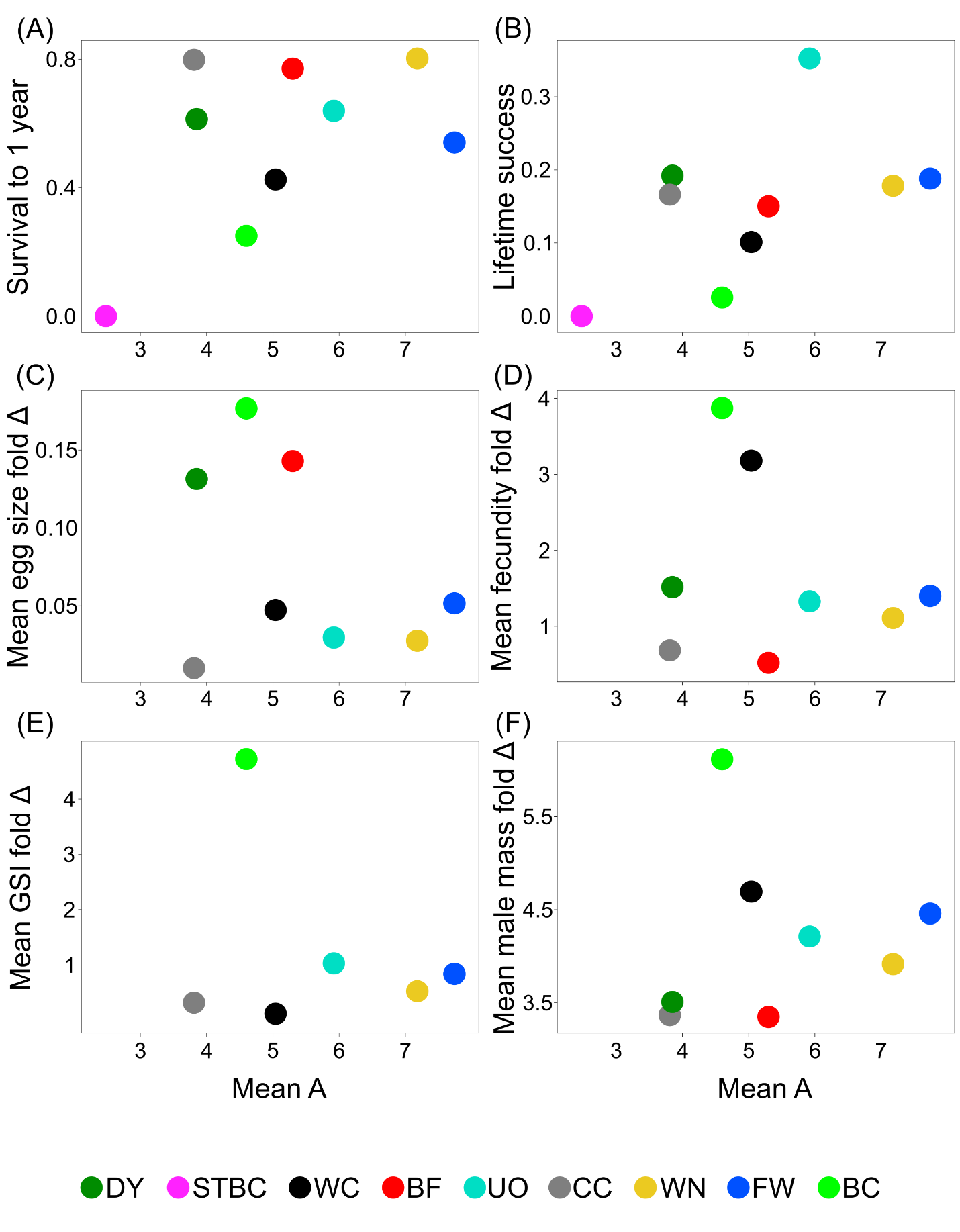


Figure A9. Relationship between allelic diversity (‘Mean A’) of different wild Cape Race brook trout populations using microsatellite loci and (A) mean captive-born survival to one year, (B) lifetime success in captivity, and the extent of phenotypic change experienced in captivity at different traits (C-F). Based on 2011 crosses.

Table A1. Total captive-born families and numbers of males and females from wild Cape Race brook trout populations used in crosses and pooled in sets for captive-rearing to adult stages.

| **Population** | **Year** | **Total families** | **No. of males** | **No. of females** | **Set males** | **Set females** |
| --- | --- | --- | --- | --- | --- | --- |
| Bob’s Cove (BC) | 2011 | 29 | 11 | 9 | 11 | 9 |
|  | 2014 | 10 | 7 | 5 | NA | NA |
| Blackfly (BF) | 2011 | 31 | 15 | 12 | 15 | 12 |
|  | 2014 | NA | NA | NA | NA | NA |
| Cripple Cove (CC) | 2011 | 49 | 17 | 12 | 17 | 12 |
|  | 2014 | 29 | 10 | 4 | NA | NA |
| Ditchy (DY) | 2011 | 17 | 14 | 6 | 12 | 6 |
|  | 2014 | NA | NA | NA | NA | NA |
| Freshwater (FW) | 2011 | 60 | 22 | 30 | 19 | 12 |
|  | 2014 | 65 | 20 | 33 | 19 | 12 |
| Still There By Chance (STBC) | 2011 | 41 | 18 | 14 | 17 | 12 |
|  | 2014 | 11 | 10 | 7 | 10 | 7 |
| Upper Coquita (UC) | 2011 | NA | NA | NA | NA | NA |
|  | 2014 | 13 | 10 | 7 | NA | NA |
| Upper O’Beck (UO) | 2011 | 64 | 30 | 29 | 19 | 12 |
|  | 2014 | 67 | 30 | 33 | 19 | 12 |
| Whale Cove (WC) | 2011 | 49 | 17 | 19 | 17 | 12 |
|  | 2014 | 18 | 17 | 10 | 17 | 10 |
| Watern (WN) | 2011 | 49 | 15 | 19 | 15 | 12 |
|  | 2014 | 52 | 20 | 22 | 19 | 12 |

Table A2. Population genetic and genomic data from wild Cape Race brook trout populations used for analyses on the extent of plastic and genetic change experienced in a common hatchery environment, including the mean census population size (*N_c_*), mean effective number of breeders (*N_b_*), heterozygosity (H_o_) and allelic diversity (number of alleles/locus, A_r_) based on 5 to 7 year averages derived from 12 microsatellites (Bernos & Fraser 2016), and heterozygosity (H_o_) based on 164 neutral SNPs from Fraser et al. (2014).

| Population | N_c_ | N_b_ | H_O_ | A | H_O_ |
| --- | --- | --- | --- | --- | --- |
| DY | 116 | 10 | 0.54 | 3.85 | 0.160 |
| WC | 783 | 31 | 0.52 | 5.04 | 0.196 |
| STBC | 917 | 28 | 0.27 | 2.48 | 0.062 |
| BF | 1184 | 52 | 0.54 | 5.30 | 0.226 |
| CC | 1862 | 74 | 0.43 | 3.81 | 0.162 |
| UO | 2569 | 62 | 0.60 | 5.92 | 0.180 |
| BC | 4693 | 355 | 0.46 | 4.60 | 0.110 |
| WN | 7801 | 178 | 0.57 | 7.18 | 0.235 |
| FW | 5307 | 207 | 0.61 | 7.74 | 0.240 |

Table A3. Mean slope estimates for growth rate for wild Cape Race brook trout populations reared in a common hatchery environment. Ln-transformed mass reflects specific growth rate.

|  | Length (mm/month) | | Mass ln(g)/month | |
| --- | --- | --- | --- | --- |
| Population | Slope | S.E. | Slope | S.E. |
| BF | 7.455 | 0.127 | 0.289 | 0.005 |
| CC | 9.352 | 0.115 | 0.302 | 0.004 |
| DY | 7.639 | 0.117 | 0.292 | 0.005 |
| FW | 6.407 | 0.112 | 0.259 | 0.005 |
| OB | 7.198 | 0.118 | 0.259 | 0.004 |
| WC | 9.110 | 0.095 | 0.327 | 0.005 |
| WN | 7.497 | 0.111 | 0.284 | 0.005 |

Table A4. Pairwise comparisons of specific growth rates for length and mass between Cape Race brook trout populations reared in a common hatchery environment.

|  | Length (mm/month) | | |  |  | Mass (ln(g)/month) | |  |  |  |
| --- | --- | --- | --- | --- | --- | --- | --- | --- | --- | --- |
| Contrast | Estimate | S.E. | df | *t*-ratio | p | Estimate | S.E. | df | *t*-ratio | p |
| BF – CC | -1.897 | 0.193 | 53.04 | -9.847 | <0.001 | -0.013 | 0.007 | 300.94 | -2.011 | 0.949 |
| BF – DY | -0.184 | 0.173 | 75.64 | -1.065 | 1.000 | -0.003 | 0.007 | 428.89 | -0.459 | 1.000 |
| BF – FW | 1.048 | 0.180 | 47.25 | 5.822 | <0.001 | 0.030 | 0.007 | 244.79 | 4.385 | <0.001 |
| BF – UO | 0.257 | 0.183 | 48.57 | 1.408 | 1.000 | 0.030 | 0.007 | 289.09 | 4.473 | <0.001 |
| BF – WC | -1.655 | 0.159 | 63.46 | -10.405 | <0.001 | -0.038 | 0.007 | 345.44 | -5.326 | <0.001 |
| BF – WN | -0.042 | 0.169 | 57.42 | -0.249 | 1.000 | 0.005 | 0.007 | 304.25 | 0.782 | 1.000 |
| CC – DY | 1.713 | 0.164 | 54.38 | 10.425 | <0.001 | 0.010 | 0.007 | 376.65 | 1.461 | 1.000 |
| CC – FW | 2.945 | 0.178 | 31.35 | 16.574 | <0.001 | 0.044 | 0.006 | 207.09 | 6.891 | <0.001 |
| CC – UO | 2.154 | 0.182 | 31.97 | 11.829 | <0.001 | 0.043 | 0.006 | 245.06 | 7.107 | <0.001 |
| CC – WC | 0.242 | 0.150 | 44.41 | 1.614 | 1.000 | -0.025 | 0.007 | 298.92 | -3.786 | 0.004 |
| CC – WN | 1.855 | 0.160 | 39.08 | 11.563 | <0.001 | 0.019 | 0.006 | 258.04 | 2.998 | 0.063 |
| DY – FW | 1.232 | 0.162 | 48.46 | 7.597 | <0.001 | 0.034 | 0.007 | 308.64 | 4.756 | <0.001 |
| DY – UO | 0.441 | 0.166 | 49.85 | 2.653 | 0.225 | 0.033 | 0.007 | 362.49 | 4.851 | <0.001 |
| DY – WC | -1.471 | 0.151 | 64.9 | -9.748 | <0.001 | -0.035 | 0.007 | 421.01 | -4.753 | <0.001 |
| DY – WN | 0.142 | 0.192 | 58.81 | 0.740 | 1.000 | 0.009 | 0.007 | 380.87 | 1.246 | 1.000 |
| FW – UO | -0.790 | 0.171 | 28.17 | -4.613 | 0.002 | 0.000 | 0.006 | 199.35 | -0.061 | 1.000 |
| FW – WC | -2.703 | 0.147 | 39.48 | -18.341 | <0.001 | -0.069 | 0.007 | 245.14 | -9.864 | <0.001 |
| FW – WN | -1.090 | 0.158 | 34.56 | -6.890 | <0.001 | -0.025 | 0.007 | 208.99 | -3.823 | 0.004 |
| UO - WC | -1.913 | 0.152 | 40.44 | -12.583 | <0.001 | -0.068 | 0.007 | 287.62 | -10.133 | <0.001 |
| UO - WN | -0.299 | 0.162 | 35.32 | -1.842 | 1.000 | -0.025 | 0.006 | 247.61 | -3.905 | 0.002 |
| WC - WN | 1.613 | 0.147 | 48.34 | 10.998 | <0.001 | 0.044 | 0.007 | 302.07 | 6.382 | <0.001 |

* *p-*values are Bonferroni-adjusted.

Table A5. Pairwise comparisons of mean population length (mm) at 0 and 12 months among Cape Race brook trout populations reared in a common hatchery environment. Diff. = difference

|  | 0 Months |  |  |  |  | 12 Months |  |  |  |  |
| --- | --- | --- | --- | --- | --- | --- | --- | --- | --- | --- |
| Contrast | Diff. (mm) | S.E. | df | *t*-ratio | p* | Diff. (mm) | S.E. | df | *t*-ratio | p* |
| BF – CC | 6.663 | 0.744 | 98.32 | 8.956 | <0.001 | -16.102 | 1.894 | 30.72 | -8.504 | <0.001 |
| BF – DY | 3.659 | 1.724 | 88.11 | 2.123 | 0.769 | 1.451 | 2.456 | 30.9 | 0.591 | 1.000 |
| BF – FW | 1.638 | 0.599 | 147.51 | 2.733 | 0.147 | 14.210 | 1.843 | 31.1 | 7.710 | <0.001 |
| BF – UO | 3.771 | 0.720 | 42.39 | 5.238 | <0.001 | 6.857 | 1.949 | 30.68 | 3.518 | 0.029 |
| BF – WC | 3.207 | 2.174 | 32.76 | 1.475 | 1.000 | -16.657 | 2.744 | 30.86 | -6.071 | <0.001 |
| BF – WN | 1.955 | 1.724 | 69.23 | 1.134 | 1.000 | 1.450 | 2.420 | 30.79 | 0.599 | 1.000 |
| CC – DY | -3.004 | 1.716 | 52.12 | -1.751 | 1.000 | 17.553 | 2.381 | 30.33 | 7.373 | <0.001 |
| CC – FW | -5.025 | 0.697 | 24.34 | -7.206 | <0.001 | 30.312 | 1.798 | 30.46 | 16.855 | <0.001 |
| CC – UO | -2.893 | 0.536 | 35.16 | -5.399 | <0.001 | 22.960 | 1.878 | 29.98 | 12.228 | <0.001 |
| CC – WC | -3.456 | 2.168 | 18.15 | -1.595 | 1.000 | -0.555 | 2.676 | 30.3 | -0.207 | 1.000 |
| CC – WN | -4.708 | 1.716 | 38.35 | -2.744 | 0.193 | 17.552 | 2.343 | 30.12 | 7.491 | <0.001 |
| DY – FW | -2.021 | 1.705 | 44.55 | -1.186 | 1.000 | 12.760 | 2.387 | 30.7 | 5.345 | <0.001 |
| DY – UO | 0.111 | 1.702 | 40.81 | 0.065 | 1.000 | 5.407 | 2.447 | 30.3 | 2.210 | 0.731 |
| DY – WC | -0.452 | 2.342 | 32 | -0.193 | 1.000 | -18.108 | 2.881 | 30.55 | -6.286 | <0.001 |
| DY – WN | -1.704 | 0.480 | 501.58 | -3.550 | 0.008 | -0.001 | 1.960 | 30.24 | 0.000 | 1.000 |
| FW – UO | 2.132 | 0.569 | 31.64 | 3.745 | 0.015 | -7.353 | 1.799 | 30.38 | -4.086 | 0.006 |
| FW – WC | 1.569 | 2.159 | 15.36 | 0.727 | 1.000 | -30.867 | 2.682 | 30.68 | -11.510 | <0.001 |
| FW – WN | 0.317 | 1.705 | 32.21 | 0.186 | 1.000 | -12.760 | 2.350 | 30.55 | -5.431 | <0.001 |
| UO – WC | -0.564 | 2.156 | 13.74 | -0.261 | 1.000 | -23.515 | 2.735 | 30.29 | -8.598 | <0.001 |
| UO – WN | -1.815 | 1.702 | 29.04 | -1.067 | 1.000 | -5.407 | 2.410 | 30.09 | -2.244 | 0.680 |
| WC - WN | -1.252 | 2.342 | 23.41 | -0.534 | 1.000 | 18.107 | 2.850 | 30.35 | 6.354 | <0.001 |
|  |  |  |  |  |  |  |  |  |  |  |

* *p-*values are Bonferroni-adjusted.

Table A6. Pairwise comparisons of mean population mass (ln(g)) at 3 and 12 months among Cape Race brook trout populations reared in a common hatchery environment. Diff. = difference

|  | 3 Months |  |  |  |  | 12 Months | |  |  |  |
| --- | --- | --- | --- | --- | --- | --- | --- | --- | --- | --- |
| Contrast | Diff. (ln(g)) | S.E. | df | *t*-ratio | p* | Diff. (ln(g)) | S.E. | df | *t*-ratio | p* |
| BF – CC | -0.175 | 0.033 | 16.64 | -5.351 | 0.002 | -0.294 | 0.051 | 26.49 | -5.741 | <0.001 |
| BF – DY | 0.157 | 0.034 | 23.58 | 4.679 | 0.002 | 0.127 | 0.059 | 27.83 | 2.159 | 0.832 |
| BF – FW | 0.103 | 0.034 | 16.45 | 3.078 | 0.147 | 0.376 | 0.055 | 26.89 | 6.888 | <0.001 |
| BF – UO | -0.125 | 0.031 | 16.51 | -4.082 | 0.017 | 0.144 | 0.054 | 26.56 | 2.652 | 0.279 |
| BF – WC | -0.121 | 0.034 | 16.49 | -3.553 | 0.053 | -0.466 | 0.057 | 28.29 | -8.186 | <0.001 |
| BF – WN | -0.015 | 0.031 | 16.53 | -0.480 | 1.000 | 0.033 | 0.055 | 26.45 | 0.597 | 1.000 |
| CC – DY | 0.332 | 0.034 | 23.93 | 9.630 | <0.001 | 0.421 | 0.053 | 26.89 | 8.011 | <0.001 |
| CC – FW | 0.279 | 0.035 | 16.7 | 8.073 | <0.001 | 0.670 | 0.048 | 25.69 | 13.991 | <0.001 |
| CC – UO | 0.050 | 0.032 | 16.75 | 1.575 | 1.000 | 0.438 | 0.048 | 25.35 | 9.216 | <0.001 |
| CC – WC | 0.055 | 0.035 | 16.74 | 1.568 | 1.000 | -0.171 | 0.051 | 27.42 | -3.392 | 0.044 |
| CC – WN | 0.160 | 0.032 | 16.77 | 4.984 | 0.002 | 0.327 | 0.049 | 25.23 | 6.746 | <0.001 |
| DY – FW | -0.054 | 0.035 | 23.69 | -1.524 | 1.000 | 0.249 | 0.056 | 27.28 | 4.466 | 0.002 |
| DY – UO | -0.282 | 0.033 | 23.77 | -8.672 | <0.001 | 0.017 | 0.056 | 26.96 | 0.309 | 1.000 |
| DY – WC | -0.278 | 0.036 | 23.65 | -7.792 | <0.001 | -0.592 | 0.058 | 28.6 | -10.204 | <0.001 |
| DY – WN | -0.172 | 0.033 | 23.8 | -5.214 | <0.001 | -0.094 | 0.056 | 26.85 | -1.667 | 1.000 |
| FW – UO | -0.229 | 0.033 | 16.56 | -7.021 | <0.001 | -0.232 | 0.051 | 25.77 | -4.540 | 0.002 |
| FW – WC | -0.224 | 0.036 | 16.55 | -6.283 | <0.001 | -0.842 | 0.054 | 27.81 | -15.622 | <0.001 |
| FW – WN | -0.118 | 0.033 | 16.58 | -3.585 | 0.050 | -0.343 | 0.052 | 26.65 | -6.598 | <0.001 |
| UO – WC | 0.005 | 0.033 | 16.6 | 0.143 | 1.000 | -0.610 | 0.054 | 27.48 | -11.381 | <0.001 |
| UO – WN | 0.110 | 0.030 | 16.64 | 3.670 | 0.042 | -0.111 | 0.052 | 25.3 | -2.149 | 0.869 |
| WC – WN | 0.106 | 0.033 | 16.62 | 3.165 | 0.122 | 0.499 | 0.054 | 27.38 | 9.163 | <0.001 |

* *p-*values are Bonferroni-adjusted.

Table A7. Pairwise comparisons of mean population length (mm) at 18 months between Cape Race brook trout populations reared in a common hatchery environment.

| Contrast | Difference (mm) | S.E. | df | *t*-ratio | p* |
| --- | --- | --- | --- | --- | --- |
| BC – BF | -6.641 | 6.654 | 840 | -0.998 | 1.000 |
| BC – CC | -33.740 | 6.104 | 840 | -5.527 | <0.001 |
| BC – DY | 3.522 | 6.365 | 840 | 0.553 | 1.000 |
| BC – FW | 20.536 | 6.191 | 840 | 3.317 | 0.027 |
| BC – UO | -3.465 | 6.276 | 840 | -0.552 | 1.000 |
| BC – WC | -46.224 | 7.317 | 840 | -6.317 | <0.001 |
| BC – WN | -5.772 | 6.283 | 840 | -0.919 | 1.000 |
| BF – CC | -27.100 | 3.201 | 840 | -8.465 | <0.001 |
| BF – DY | 10.163 | 3.645 | 840 | 2.788 | 0.152 |
| BF – FW | 27.177 | 3.362 | 840 | 8.083 | <0.001 |
| BF – UO | 3.176 | 3.512 | 840 | 0.904 | 1.000 |
| BF – WC | -39.583 | 5.116 | 840 | -7.737 | <0.001 |
| BF – WN | 0.869 | 3.520 | 840 | 0.247 | 1.000 |
| CC – DY | 37.262 | 2.536 | 840 | 14.693 | <0.001 |
| CC – FW | 54.277 | 2.090 | 840 | 25.969 | <0.001 |
| CC – UO | 30.276 | 2.326 | 840 | 13.018 | <0.001 |
| CC – WC | -12.483 | 4.403 | 840 | -2.836 | 0.131 |
| CC – WN | 27.969 | 2.341 | 840 | 11.945 | <0.001 |
| DY – FW | 17.014 | 2.736 | 840 | 6.219 | <0.001 |
| DY – UO | -6.986 | 2.916 | 840 | -2.396 | 0.470 |
| DY – WC | -49.746 | 4.716 | 840 | -10.548 | <0.001 |
| DY – WN | -9.293 | 2.924 | 840 | -3.178 | 0.043 |
| FW – UO | -24.001 | 2.543 | 840 | -9.437 | <0.001 |
| FW – WC | -66.760 | 4.520 | 840 | -14.769 | <0.001 |
| FW – WN | -26.308 | 2.558 | 840 | -10.286 | <0.001 |
| UO – WC | -42.759 | 4.630 | 840 | -9.235 | <0.001 |
| UO – WN | -2.307 | 2.752 | 840 | -0.838 | 1.000 |
| WC - WN | 40.452 | 4.633 | 840 | 8.731 | <0.001 |

* *p-*values are Bonferroni-adjusted.

Table A8. Pairwise comparisons of mean population mass (g) at 18 months, by sex, among Cape Race trout populations reared in a common hatchery environment. Diff. = difference.

|  | Females |  |  |  |  | Males |  |  |  |  |
| --- | --- | --- | --- | --- | --- | --- | --- | --- | --- | --- |
| Contrast | Diff. (g) | S.E. | df | *t*-ratio | p* | Diff. (g) | S.E. | df | *t*-ratio | p* |
| BC – BF | 0.890 | 13.679 | 833 | 0.065 | 1.000 | -19.391 | 15.542 | 833 | -1.248 | 1.000 |
| BC – CC | -43.989 | 11.592 | 833 | -3.795 | 0.004 | -47.693 | 14.643 | 833 | -3.257 | 0.033 |
| BC – DY | 22.527 | 12.768 | 833 | 1.764 | 1.000 | -4.119 | 14.811 | 833 | -0.278 | 1.000 |
| BC – FW | 34.509 | 11.407 | 833 | 3.025 | 0.072 | 29.367 | 14.499 | 833 | 2.025 | 1.000 |
| BC – UO | 15.609 | 11.829 | 833 | 1.320 | 1.000 | -14.013 | 14.743 | 833 | -0.951 | 1.000 |
| BC – WC | -101.596 | 22.203 | 833 | -4.576 | <0.001 | -69.213 | 16.638 | 833 | -4.160 | 0.001 |
| BC – WN | 6.992 | 12.104 | 833 | 0.578 | 1.000 | -13.597 | 14.843 | 833 | -0.916 | 1.000 |
| BF – CC | -44.879 | 8.857 | 833 | -5.067 | <0.001 | -28.302 | 7.134 | 833 | -3.967 | 0.002 |
| BF – DY | 21.637 | 10.350 | 833 | 2.091 | 1.000 | 15.272 | 7.472 | 833 | 2.044 | 1.000 |
| BF – FW | 33.619 | 8.614 | 833 | 3.903 | 0.003 | 48.758 | 6.834 | 833 | 7.135 | <0.001 |
| BF – UO | 14.719 | 9.166 | 833 | 1.606 | 1.000 | 5.377 | 7.336 | 833 | 0.733 | 1.000 |
| BF – WC | -102.486 | 20.906 | 833 | -4.902 | <0.001 | -49.822 | 10.644 | 833 | -4.681 | <0.001 |
| BF – WN | 6.102 | 9.518 | 833 | 0.641 | 1.000 | 5.794 | 7.535 | 833 | 0.769 | 1.000 |
| CC – DY | 66.515 | 7.373 | 833 | 9.022 | <0.001 | 43.574 | 5.358 | 833 | 8.132 | <0.001 |
| CC – FW | 78.498 | 4.630 | 833 | 16.955 | <0.001 | 77.060 | 4.424 | 833 | 17.418 | <0.001 |
| CC – UO | 59.597 | 5.590 | 833 | 10.661 | <0.001 | 33.679 | 5.166 | 833 | 6.519 | <0.001 |
| CC – WC | -57.607 | 19.604 | 833 | -2.939 | 0.095 | -21.520 | 9.283 | 833 | -2.318 | 0.579 |
| CC – WN | 50.980 | 6.151 | 833 | 8.288 | <0.001 | 34.096 | 5.445 | 833 | 6.261 | <0.001 |
| DY – FW | 11.983 | 7.078 | 833 | 1.693 | 1.000 | 33.486 | 4.952 | 833 | 6.762 | <0.001 |
| DY – UO | -6.918 | 7.741 | 833 | -0.894 | 1.000 | -9.894 | 5.625 | 833 | -1.759 | 1.000 |
| DY – WC | -124.122 | 20.322 | 833 | -6.108 | <0.001 | -65.094 | 9.546 | 833 | -6.819 | <0.001 |
| DY – WN | -15.535 | 8.155 | 833 | -1.905 | 1.000 | -9.478 | 5.882 | 833 | -1.611 | 1.000 |
| FW – UO | -18.901 | 5.196 | 833 | -3.638 | 0.008 | -43.381 | 4.744 | 833 | -9.145 | <0.001 |
| FW – WC | -136.105 | 19.495 | 833 | -6.981 | <0.001 | -98.580 | 9.055 | 833 | -10.887 | <0.001 |
| FW – WN | -27.518 | 5.795 | 833 | -4.748 | <0.001 | -42.964 | 5.046 | 833 | -8.514 | <0.001 |
| UO – WC | -117.204 | 19.745 | 833 | -5.936 | <0.001 | -55.200 | 9.440 | 833 | -5.848 | <0.001 |
| UO – WN | -8.617 | 6.588 | 833 | -1.308 | 1.000 | 0.417 | 5.708 | 833 | 0.073 | 1.000 |
| WC – WN | 108.587 | 19.911 | 833 | 5.454 | <0.001 | 55.616 | 9.595 | 833 | 5.796 | <0.001 |

* *p-*values are Bonferroni-adjusted.

Table A9. Pairwise comparisons of mean population fecundity among Cape Race brook trout

populations in a common hatchery environment.

| Contrast | Difference | S.E. | | df | *t*-ratio | p* |
| --- | --- | --- | --- | --- | --- | --- |
| BC – BF | 38.718 | 25.851 | 346 | | 1.498 | 1.000 |
| BC – CC | -21.884 | 22.837 | 346 | | -0.958 | 1.000 |
| BC – DY | 68.133 | 24.750 | 346 | | 2.753 | 0.1742 |
| BC – FW | 96.965 | 22.784 | 346 | | 4.256 | 0.0008 |
| BC – UO | 73.143 | 22.914 | 346 | | 3.192 | 0.0432 |
| BC – WC | -117.671 | 33.403 | 346 | | -3.523 | 0.0136 |
| BC – WN | 55.494 | 23.048 | 346 | | 2.408 | 0.4641 |
| BF – CC | -60.602 | 16.460 | 346 | | -3.682 | 0.0075 |
| BF – DY | 29.415 | 19.026 | 346 | | 1.546 | 1.000 |
| BF – FW | 58.247 | 16.386 | 346 | | 3.555 | 0.0121 |
| BF – UO | 34.425 | 16.567 | 346 | | 2.078 | 1.000 |
| BF – WC | -156.390 | 29.414 | 346 | | -5.317 | <0.0001 |
| BF – WN | 16.776 | 16.752 | 346 | | 1.001 | 1.000 |
| CC – DY | 90.017 | 14.671 | 346 | | 6.136 | <0.0001 |
| CC – FW | 118.849 | 11.034 | 346 | | 10.771 | <0.0001 |
| CC – UO | 95.0266 | 11.301 | 346 | | 8.408 | <0.0001 |
| CC – WC | -95.788 | 26.803 | 346 | | -3.574 | 0.0113 |
| CC – WN | 77.376 | 11.570 | 346 | | 6.688 | <0.0001 |
| DY – FW | 28.832 | 14.588 | 346 | | 1.976 | 1.000 |
| DY – UO | 5.010 | 14.791 | 346 | | 0.339 | 1.000 |
| DY – WC | -185.804 | 28.452 | 346 | | -6.531 | <0.0001 |
| DY – WN | -12.640 | 14.98 | 346 | | -0.843 | 1.000 |
| FW – UO | -23.822 | 11.194 | 346 | | -2.128 | 0.953 |
| FW – WC | -214.636 | 26.758 | 346 | | -8.021 | <0.0001 |
| FW – WN | -41.471 | 11.465 | 346 | | -3.617 | 0.0096 |
| UO – WC | -190.814 | 26.870 | 346 | | -7.102 | <0.0001 |
| UO – WN | -17.649 | 11.723 | 346 | | -1.506 | 1.000 |
| WC – WN | 173.165 | 26.983 | 346 | | 6.417 | <0.0001 |

* *p-*values are Bonferroni-adjusted.

Table A10. Pairwise comparisons of mean egg diameter among Cape Race brook trout populations in a common hatchery environment.

| Contrast | Difference (mm) | S.E. | df | *t*-ratio | p* |
| --- | --- | --- | --- | --- | --- |
| BC – BF | 38.718 | 25.851 | 346 | 1.498 | 1.000 |
| BC – CC | -21.884 | 22.837 | 346 | -0.958 | 1.000 |
| BC – DY | 68.133 | 24.750 | 346 | 2.753 | 0.1742 |
| BC - FW | 96.965 | 22.784 | 346 | 4.256 | 0.0008 |
| BC - UO | 73.143 | 22.914 | 346 | 3.192 | 0.0432 |
| BC - WC | -117.671 | 33.403 | 346 | -3.523 | 0.0136 |
| BC - WN | 55.494 | 23.048 | 346 | 2.408 | 0.4641 |
| BF - CC | -60.602 | 16.460 | 346 | -3.682 | 0.0075 |
| BF - DY | 29.415 | 19.026 | 346 | 1.546 | 1.000 |
| BF - FW | 58.247 | 16.386 | 346 | 3.555 | 0.0121 |
| BF – UO | 34.425 | 16.567 | 346 | 2.078 | 1.000 |
| BF – WC | -156.390 | 29.414 | 346 | -5.317 | <0.0001 |
| BF – WN | 16.776 | 16.752 | 346 | 1.001 | 1.000 |
| CC – DY | 90.017 | 14.671 | 346 | 6.136 | <0.0001 |
| CC – FW | 118.849 | 11.034 | 346 | 10.771 | <0.0001 |
| CC – UO | 95.0266 | 11.301 | 346 | 8.408 | <0.0001 |
| CC – WC | -95.788 | 26.803 | 346 | -3.754 | 0.0113 |
| CC – WN | 77.378 | 11.570 | 346 | 6.688 | <0.0001 |
| DY – FW | 28.832 | 14.588 | 346 | 1.976 | 1.000 |
| DY – UO | 5.010 | 14.791 | 346 | 0.339 | 1.000 |
| DY – WC | -185.805 | 28.451 | 346 | -6.531 | <0.0001 |
| DY – WN | -12.640 | 14.998 | 346 | -0.843 | 1.000 |
| FW – UO | -23.822 | 11.194 | 346 | -2.128 | 0.953 |
| FW – WC | -214.636 | 26.758 | 346 | -8.021 | <0.0001 |
| FW – WN | -41.471 | 11.465 | 346 | -3.617 | 0.0096 |
| UO – WC | -190.814 | 26.870 | 346 | -7.102 | <0.0001 |
| UO – WN | -17.649 | 11.723 | 346 | -1.506 | 1.000 |
| WC – WN | 173.165 | 26.983 | 346 | 6.417 | <0.0001 |

Table A11. Pairwise comparisons of GSI among Cape Race brook trout populations in a common hatchery environment.

| Contrast | Difference | S.E. | df | *t*-ratio | p* |
| --- | --- | --- | --- | --- | --- |
| BC - BF | 2.775 | 0.948 | 339 | 2.928 | 0.1019 |
| BC - CC | 2.401 | 0.838 | 339 | 2.864 | 0.1246 |
| BC - DY | 0.405 | 0.907 | 339 | 0.447 | 1.000 |
| BC - FW | 0.623 | 0.836 | 339 | 0.744 | 1.000 |
| BC - UO | 2.362 | 0.841 | 339 | 2.810 | 0.1467 |
| BC - WC | 3.237 | 1.283 | 339 | 2.523 | 0.3385 |
| BC - WN | 2.535 | 0.846 | 339 | 2.997 | 0.0819 |
| BF - CC | -0.374 | 0.605 | 339 | -0.617 | 1.000 |
| BF - DY | -2.369 | 0.697 | 339 | -3.397 | 0.0213 |
| BF - FW | -2.152 | 0.602 | 339 | -3.572 | 0.0113 |
| BF – UO | -0.412 | 0.608 | 339 | -0.678 | 1.000 |
| BF – WC | 0.463 | 1.144 | 339 | 0.404 | 1.000 |
| BF – WN | -0.240 | 0.615 | 339 | -0.390 | 1.000 |
| CC – DY | -1.996 | 0.540 | 339 | -3.697 | 0.0071 |
| CC – FW | -1.778 | 0.410 | 339 | -4.338 | 0.0005 |
| CC – UO | -0.039 | 0.419 | 339 | -0.092 | 1.000 |
| CC – WC | 0.836 | 1.056 | 339 | 0.792 | 1.000 |
| CC – WN | 0.134 | 0.429 | 339 | 0.312 | 1.000 |
| DY – FW | 0.217 | 0.537 | 339 | 0.405 | 1.000 |
| DY – UO | 1.957 | 0.543 | 339 | 3.602 | 0.0102 |
| DY – WC | 2.832 | 1.111 | 339 | 2.549 | 0.315 |
| DY – WN | 2.130 | 0.551 | 339 | 3.864 | 0.0037 |
| FW – UO | 1.740 | 0.414 | 339 | 4.198 | 0.001 |
| FW – WC | 2.614 | 1.054 | 339 | 2.480 | 0.381 |
| FW – WN | 1.912 | 0.425 | 339 | 4.504 | 0.0003 |
| UO – WC | 0.875 | 1.057 | 339 | 0.827 | 1.000 |
| UO – WN | 0.172 | 0.433 | 339 | 0.398 | 1.000 |
| WC – WN | -0.702 | 1.062 | 339 | -0.662 | 1.000 |

Table A12. Pairwise comparisons of mean population survival among Cape Race trout populations in a common hatchery environment, from post-yolk absorption to age one year.

| contrast | odds.ratio | SE | df | z.ratio | Padj |
| --- | --- | --- | --- | --- | --- |
| FW - STBC | 156.969 | 96.829 | 8 | 8.196 | <0.001 |
| FW - UO | 0.627 | 0.145 | 8 | -2.022 | 0.778 |
| FW - WC | 1.653 | 0.383 | 9 | 2.167 | 0.584 |
| FW - WN | 0.247 | 0.056 | 8 | -6.164 | 0.003 |
| STBC - UO | 0.004 | 0.002 | 8 | -8.931 | <0.001 |
| STBC - WC | 0.011 | 0.007 | 8 | -7.361 | <0.001 |
| STBC - WN | 0.002 | 0.001 | 9 | -10.462 | <0.001 |
| UO - WC | 2.634 | 0.619 | 8 | 4.121 | 0.033 |
| UO - WN | 0.394 | 0.091 | 9 | -4.054 | 0.029 |
| WC - WN | 0.150 | 0.035 | 9 | -8.216 | <0.001 |

* *p-*values are Bonferroni-adjusted.

Table A13. Proportion of captive females spawned within each Cape Race trout population reared in a common hatchery environment that ultimately produced poor quality, non-developing eggs. Based on 2011 crosses.

| Population | Females spawned | Females with non-developing eggs | Proportion of females with non-developing eggs |
| --- | --- | --- | --- |
| UO | 43 | 4 | 0.09 |
| DY | 24 | 6 | 0.25 |
| BC | 9 | 2 | 0.22 |
| WN | 18 | 18 | 0.50 |
| FW | 20 | 20 | 0.50 |
| WC | 8 | 4 | 0.50 |
| BF | 25 | 14 | 0.56 |
| CC | 35 | 22 | 0.63 |

Table A14. Data used for testing a positive relationship between the effective number of breeders (*N_b_*) of each wild Cape Race brook trout populations and trait CV when reared in a common hatchery environment. GSI = gonadosomatic index; FEC = fecundity; EGGS = egg size; MMASS18 = male mass at age 18 months; FMASS18 = female mass at age 18 months.

| Pop | Mean_*N_b_* | GSI_CV | FEC_CV | EGGS_CV | MMASS18_CV | FMASS18_CV |  |  |  |  |  |
| --- | --- | --- | --- | --- | --- | --- | --- | --- | --- | --- | --- |
| BC | 355 | 0.266714 | 0.527563 | 0.051305 | 0.303248 | 0.353714 |  |  |  |  |  |
| BF | 52 | 0.193823 | 0.384426 | 0.03442 | 0.285247 | 0.347128 |  |  |  |  |  |
| CC | 74 | 0.212504 | 0.307574 | 0.04019 | 0.183632 | 0.206723 |  |  |  |  |  |
| DY | 10 | 0.282091 | 0.272132 | 0.050688 | 0.320167 | 0.26964 |  |  |  |  |  |
| FW | 207 | 0.267615 | 0.429884 | 0.053706 | 0.319197 | 0.309379 |  |  |  |  |  |
| UO | 62 | 0.262471 | 0.411401 | 0.04941 | 0.304072 | 0.323778 |  |  |  |  |  |
| WC | 31 | 0.17148 | 0.352724 | 0.021902 | 0.26795 | 0.286393 |  |  |  |  |  |
| WN | 178 | 0.191263 | 0.355093 | 0.039279 | 0.348154 | 0.294903 |  |  |  |  |  |
